# Polyphenols enhance the activity of alkylating agents in leukaemia cell lines

**DOI:** 10.18632/oncotarget.27068

**Published:** 2019-07-16

**Authors:** Amani A. Mahbub, Christine L. Le Maitre, Sarah Haywood-Small, Neil A. Cross, Nicola Jordan-Mahy

**Affiliations:** ^1^ Faculty of Applied Medical Sciences, Laboratory Medicine Department, Umm Al Qura University, Makkah, Saudi Arabia; ^2^ Biomolecular Sciences Research Centre, Sheffield Hallam University, Sheffield, UK

**Keywords:** leukaemia, cisplatin, cyclophosphamide, chlorambucil, polyphenols

## Abstract

Polyphenols have been shown to sensitize solid tumours to alkylating agents such as cisplatin, and induce apoptosis and/or cell-cycle arrest. Here, we assess the effects of five polyphenols alone and in combination with three alkylating agents: cisplatin, cyclophosphamide and chlorambucil in lymphoid and myeloid leukaemia cells lines, and non-tumour control cells.

In lymphoid leukaemia cell lines there was a synergistic reduction in ATP and glutathione levels, an induction of cell cycle arrest, DNA damage and apoptosis when quercetin, apigenin, emodin and rhein were combined with cisplatin and cyclophosphamide; and when apigenin and rhein were combined with chlorambucil. In myeloid leukaemia cells quercetin, apigenin and emodin showed a similar synergistic effect with all alkylating agents; however antagonistic effects were observed with some or all alkylating agents when combined with emodin, rhein and cis-stilbene. All synergistic effects were associated with reduced glutathione levels, DNA damage and apoptosis; whilst during antagonism the reverse effects were observed.

The combination of alkylating agents, particularly cisplatin with polyphenols could be promising for the treatment of lymphoid leukaemias, with apigenin showing the greatest effects. Likewise in myeloid cells apigenin also synergised the action of all alkylating agents, suggesting that apigenin may also be beneficial in myeloid leukaemias.

## INTRODUCTION

Leukaemia affects millions of people worldwide each year and mortality rates are high, despite advances in chemotherapy treatments [1, 2]. Alkylating agents such as cisplatin, cyclophosphamide and chlorambucil are major chemotherapeutic agents used for the treatment of leukaemia [1, 2]. Mechanistically, these agents form DNA cross-links, resulting in inhibition of DNA synthesis, cell cycle arrest and induction of apoptosis [3, 5, 6]. Unfortunately, the use of alkylating agents is commonly associated with many side effects including: nausea, vomiting, hearing loss, nephrotoxicity, myelosuppression and immunosuppression, all of which commonly occur in leukaemia patients [2–8].

Furthermore, alkylating agents are themselves mutagenic, teratogenic and carcinogenic; with cyclophosphamide use, being linked to an increased risk of acute myeloid leukaemia, skin and bladder cancer [2, 3, 9, 10]. Drug resistance is also a major problem, which results from increased DNA repair mechanisms; a decrease in cell permeability and/or an increase in glutathione levels, which subsequently protects against DNA damage [2, 6, 8]. Thus, systemic toxicity and drug resistance are major problems in limiting the effect of alkylating agents. For this reason, novel strategies and therapeutics for leukaemia are urgently needed.

Our previous work has demonstrated that bioactive compounds such as polyphenols (quercetin, apigenin, emodin, rhein, and cis-stilbene) show promise as anti-cancer agents in leukaemia cells lines [11], whilst having a protective effect on normal haematopoietic cells. These selected polyphenols are representatives of the major classification of polyphenols, and are commonlly found in fruits (*e.g.* apples, blueberries and grapes) and vegetables (*e.g.* onions, broccoli, and rhubarb) [12–14]. These polyphenols are also associated with improved quality of life [15] and improved outcomes in cancer patients [16].

Furthermore, our prior work demonstrated polyphenols synergistically enhanced the action of topoisomerase inhibitor agents (doxorubicin and etoposide), reducing ATP levels and inducing apoptosis in lymphoid and myeloid leukaemia cell lines; whilst protecting normal hematopoietic stem cells [17]. Anti-tumour actions of alkylating agents such as cisplatin are reported to be potentiated by polyphenols in solid tumour cell lines [18–24], and evidence to date generally support the notion that polyphenols promote the pro-apoptotic activity of alkylating agents.

Thus, this study investigated whether the most potent anti-proliferative and pro-apoptotic polyphenols (quercetin, apigenin, emodin, and cis- stilbene) [11, 17] synergistically enhance the actions of alkylating agents (cisplatin, cyclophosphamide and chlorambucil) in leukaemia cell lines. Effects on ATP levels, apoptosis and cell cycle progression were measured in lymphoid and myeloid leukaemia cell lines and two normal hematopoietic cells. Furthermore, potential mechanisms of action of combination treatments were investigated by determining caspase 8 and 9 activity, glutathione levels, and DNA damage.

## RESULTS

### The effect of alkylating agents alone on ATP levels and caspase 3 activity

Cisplatin, cyclophosphamide and chlorambucil decreased ATP levels as a marker of cellular activity and viability, and increased caspase 3 activity as a marker of apoptotic signalling in all cell lines in a dose-dependent manner compared to non-tumour haematopoietic cells (Supplementary Figure 1). The lowest significant dose (LSD) and IC50 doses at which ATP levels were reduced at 24 h in response to cisplatin, cyclophosphamide and chlorambucil alone, differed between cell lines (Table 1).

**Table 1:** The lowest significant dose (LSDs) and IC_50_ doses of cisplatin (CSP), cyclophosphamide (CYCLO) and chlorambucil (CLB) which reduced ATP levels and increased caspase 3 activity (CASP 3) when compared to the vehicle control (*P* ≤ 0.05) in two lymphoid (Jurkat and CCRF-CEM) and two myeloid (THP1 and KG-1a) leukaemia cell lines; and two non-tumour control hematopoietic stem cells (CD34+ HSCs and CD133+ HSCs)

Cell type		Doses	CSP		CYCLO		CLB	
			ATP	CASP 3	ATP	CASP 3	ATP	CASP 3
Lymphoid Cells	Jurkat	LSD	0.01 μM	0.01 μM	2 μM	10 μM	10 μM	10 μM
		IC_50_	0.4 μM	4.5 μM	>50 μM	50 μM	>50 μM	>50 μM
	CCRF-CEM	LSD	2 μM	0.4 μM	10 μM	10 μM	0.01 μM	10 μM
		IC_50_	15 μM	6 μM	>50 μM	>50 μM	6.5 μM	>50 μM
Myeloid Cells	THP1	LSD	0.01 μM	0.01 μM	0.4 μM	2 μM	0.4 μM	0.4 μM
		IC_50_	5.5 μM	5 μM	>50 μM	>50 μM	>50 μM	>50 μM
	KG1a	LSD	2 μM	2 μM	50 μM	50 μM	10 μM	10 μM
		IC_50_	>50 μM	>50 μM	>50 μM	>50 μM	>50 μM	>50 μM
Non-Tumor Control Cells	CD34^+^ HSCs	LSD	0.4 μM	0.4 μM	2 μM	2 μM	2 μM	10 μM
		IC_50_	10 μM	>50 μM	>50 μM	>50 μM	40 μM	>50 μM
	CD133^+^ HSCs	LSD	0.4 μM	2 μM	10 μM	10 μM	10 μM	10 μM
		IC_50_	40 μM	30 μM	>50 μM	>50 μM	>50 μM	>50 μM

The LSDs for ATP levels where used in subsequent investigation of combination treatments on cell-cycle progression, ATP and glutathione levels and DNA damage. The LSDs for caspase 3 activity were used for further investigation of apoptosis.

Treatment doses between 0.01 μM and 2 μM of cisplatin; 0.4 μM and 50 μM of cyclophosphamide; 0.01 μM and 10 μM of chlorambucil significantly reduced ATP levels after 24 h treatment in lymphoid and myeloid leukaemia cell lines, and the non-tumour control cells (*P* ≤ 0.05) (Supplementary Figure 1 and Table 1). KG1a myeloid cells were the most resistant cells, particularly to cyclophosphamide, with ATP levels significantly reduced only at a treatment dose of 50 μM (Table 1).

The LSDs for the induction of caspase 3 activity for cisplatin, cyclophosphamide and chlorambucil followed a similar pattern to the LSDs for ATP levels (Table 1). Once again, KG1a cells were the most resistant cells to cyclophosphamide (Supplementary Figure 1 and Table 1). The LSDs that significantly increased caspase 3 activity were the same, or slightly higher than those for ATP levels, this reflects the progression from a reduction of cell viability to early apoptosis (Table 1). These LSDs were subsequently used to analyse the effects of combination treatments and determine whether polyphenols had a synergistic or antagonistic effects on the activity of alkylating agents.

### The effect of combination treatments on ATP levels and caspase 3 activity

**Figure 1:**
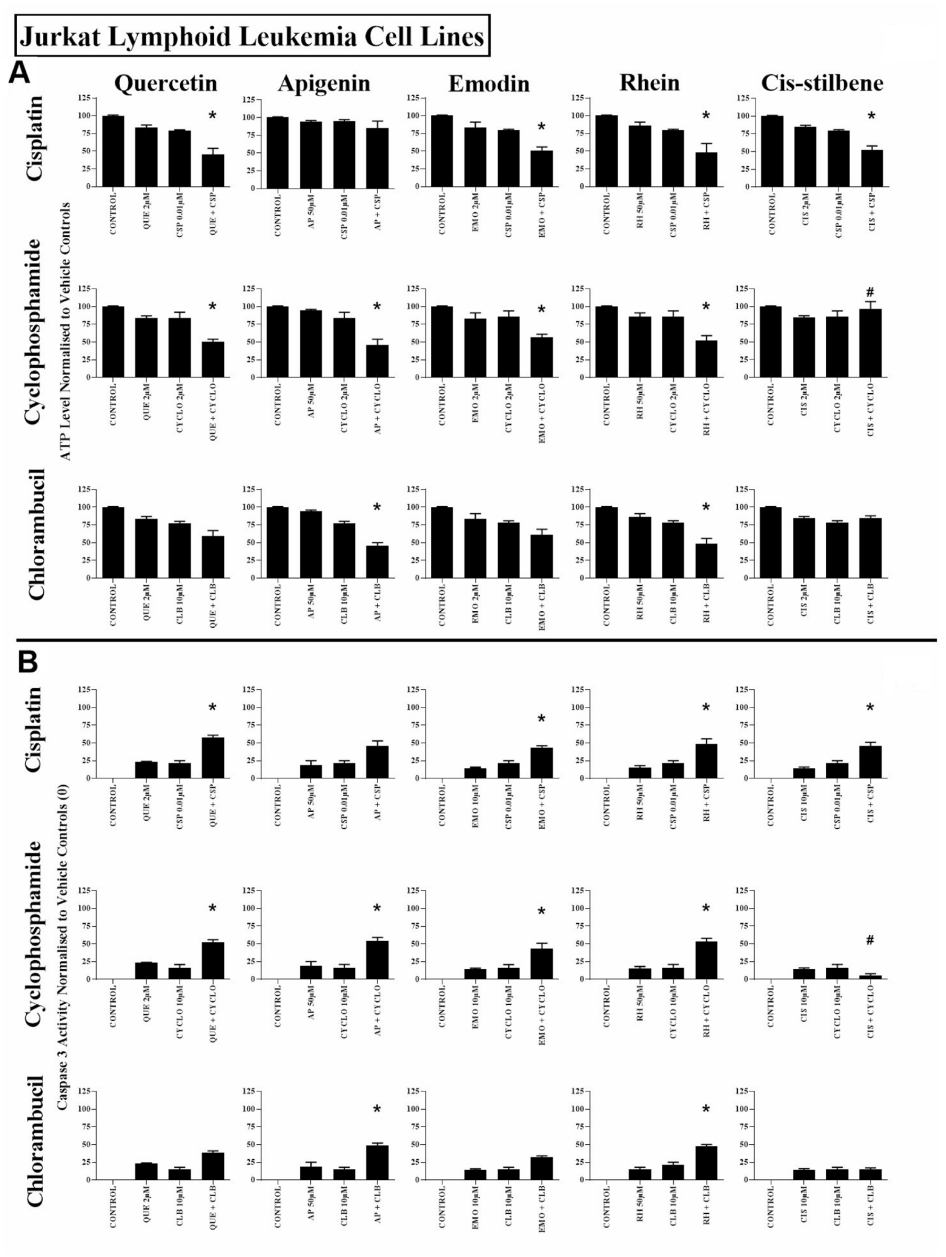
The effect of cisplatin (CSP), cyclophosphamide (CYCLO) and chlorambucil (CLB) when used in combination with quercetin (QUE), apigenin (AP), emodin (EMO), rhein (RH) or cis-stilbene (CIS) on (**A**) ATP levels and (**B**) caspase 3 activity; in the Jurkat lymphoid leukaemia cell lines. This was evaluated by: (**A**) CellTiter-Glo^®^ assay and (**B**) NucView caspase 3 activity assay. Cells were treated with CSP, CYCLO or CLB and polyphenols alone and in combination for 24 h using their lowest-significant doses (LSD); together with a vehicle control. All data was normalised to the vehicle control which was assigned (**A**) 100% cell viability and (**B**) 0% apoptosis. The data was expressed as medians and ranges (*N* = 4). Effects of combination treatments were statistically classified as synergistic (*) causing a decrease in ATP levels (**A**) and an increase in caspase 3 activity (**B**); or antagonistic (#) causing an increase in ATP levels (**A**) and a decrease in caspase 3 activity (**B**); when compared to vehicle control, drugs alone and expected values of combination treatments. Statistical significant was set at *P* ≤ 0.05.

**Figure 2:**
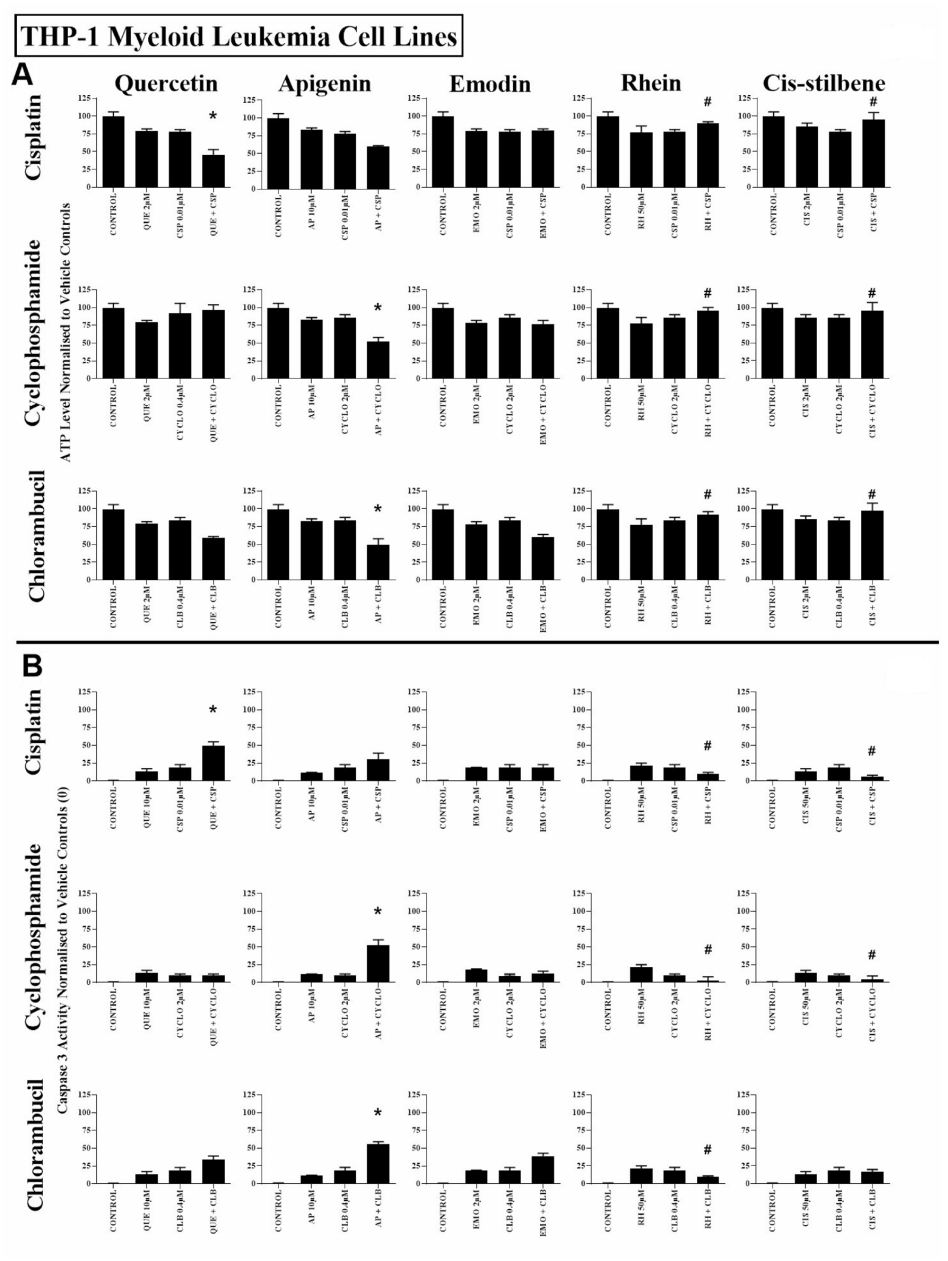
The effect of cisplatin (CSP), cyclophosphamide (CYCLO) and chlorambucil (CLB) when used in combination with quercetin (QUE), apigenin (AP), emodin (EMO), rhein (RH) or cis-stilbene (CIS) on (**A**) ATP levels and (**B**) caspase 3 activity; in the myeloid leukaemia cell line (THP-1). This was evaluated by: (**A**) CellTiter-Glo^®^ assay and (**B**) NucView caspase 3 activity assay. Cells were treated with CSP, CYCLO or CLB and polyphenols alone and in combination for 24 h using their lowest-significant doses (LSD); together with a vehicle control. All data was normalised to the vehicle control which was assigned (**A**) 100% cell viability and (**B**) 0% apoptosis. The data was expressed as medians and ranges (*N =* 4). Effects of combination treatments were statistically classified as synergistic (*) causing a decrease in ATP levels (**A**) and an increase in caspase 3 activity (**B**); or antagonistic (#) causing an increase in ATP levels (**A**) and a decrease in caspase 3 activity (**B**); when compared to vehicle control, drugs alone and expected values of combination treatments. Statistical significant was set at *P* ≤ 0.05.

All three alkylating agents significantly reduced ATP levels (Supplementary Figure 2) and induced caspase 3 activity in all leukaemia cell lines (P ≤ 0.05) (Supplementary Figure 3). However, the action of the alkylating agents was significantly affected when used in combination with polyphenols. Most notably, apigenin was shown to synergistically enhance the action of cyclophosphamide and chlorambucil; significantly decreasing ATP levels and increasing caspase 3 activity in both lymphoid and myeloid leukaemia cell lines (*P* ≤ 0.05) (Supplementary Figures 2 and 3); shown here in the Jurkat and the THP-1 leukaemia cell lines (Figures 1 and 2).

Combined treatment with quercetin and cisplatin synergistically reduced ATP levels and increased caspase 3 activity in all lymphoid and myeloid leukaemia cell lines (Supplementary Figures 2 and 3), shown here in Jurkat and THP-1 cells (Figures 1 and 2). Similarly quercetin enhanced cyclophosphamide responses in the lymphoid cell lines Jurkat (Figure 1) and CCRF-CEM (*P* ≤ 0.05) (Supplementary Figures 2 and 3). Emodin also synergistically enhanced the action of cisplatin and cyclophosphamide in all lymphoid cell lines (*P* ≤ 0.05) (Figure 1, Supplementary Figures 2 and 3), but had no significant effects in myeloid cell lines (Figure 2, Supplementary Figures 2 and 3).

Rhein was shown to synergize the action of all three alkylating agents in both lymphoid cell lines: Jurkat (Figure 1) and CCRF-CEM cells (Supplementary Figures 2 and 3), causing a significant decrease in ATP levels and an increase in caspase 3 activity (*P* ≤ 0.05). In contrast, in myeloid cell lines rhein antagonized all three alkylating agents in THP-1 cells (Figure 2), and cyclophosphamide and chlorambucil in KG-1a cells (Supplementary Figures 2 and 3); resulting in an increase in ATP levels and cell survival (*P* ≤ 0.05). Finally, *cis*-stilbene was shown to synergize only cisplatin in Jurkat (Figure 1) and CCRF-CEM cells (Supplementary Figures 2 and 3) (*P* ≤ 0.05); whilst having an antagonistic effect with cyclophosphamide in lymphoid cells (Figure 1, Supplementary Figures 2 and 3); with all three alkylating agents in THP-1 cells (Figure 2) and with chlorambucil in KG1a cells (Supplementary Figures 2 and 3) (*P* ≤ 0.05).

**Figure 3:**
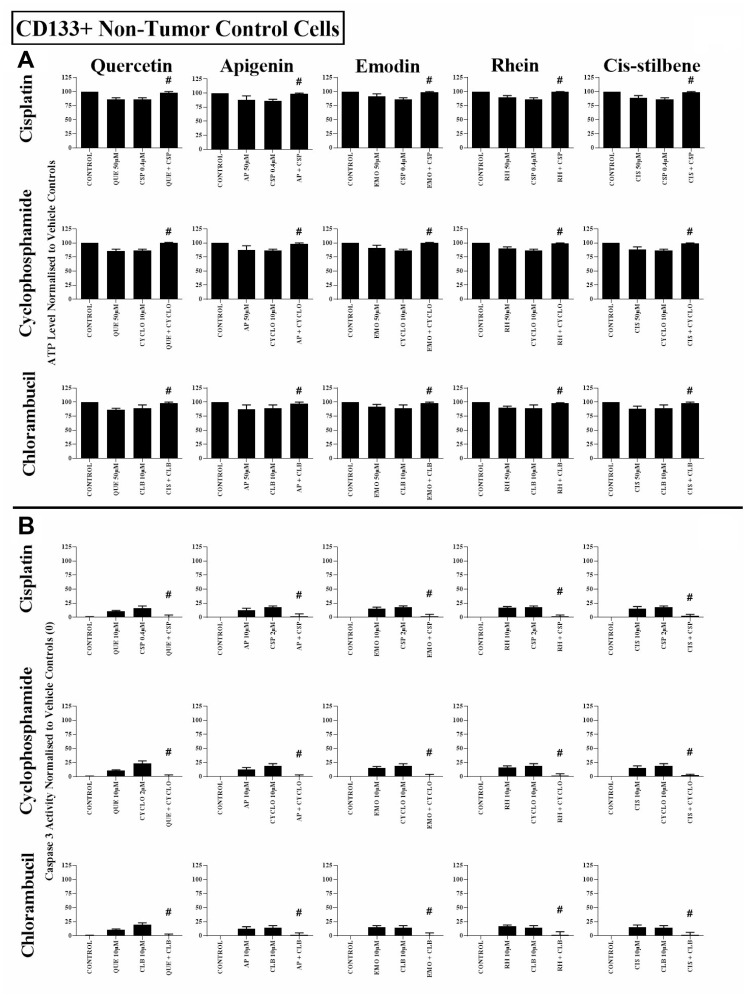
The effect of cisplatin (CSP), cyclophosphamide (CYCLO) and chlorambucil (CLB) when used in combination with quercetin (QUE), apigenin (AP), emodin (EMO), rhein (RH) or cis-stilbene (CIS) on (**A**) ATP levels and (**B**) caspase 3 activity; in non-tumour control cells (CD133+ HSC). This was evaluated by (**A**) CellTiter-Glo^®^ assay and (**B**) NucView caspase 3 activity assay Cells were treated with CSP, CYCLO or CLB and polyphenols alone and in combination for 24 h using their lowest-significant doses (LSD); together with a vehicle control. All data was normalised to the vehicle control which was assigned 100% cell viability (**A**) and 0% apoptosis (**B**). The data was expressed as medians and ranges (*N* = 4). Effects of combination treatments were statistically classified as synergistic (*) causing a decrease in ATP levels (**A**) and an increase in caspase 3 activity (**B**); or antagonistic (#) causing an increase in ATP levels (**A**) and a decrease in caspase 3 activity (**B**); when compared to vehicle control, drugs alone and expected values of combination treatments. Statistical significant was set at *P* ≤ 0.05.

In non-tumour haematopoietic control cells (CD133+HSC and CD34+HSC), all polyphenols antagonised the action of all alkylating agents, significantly increasing both ATP levels and cell survival (*P* ≤ 0.05) (Figure 3, Supplementary Figures 2 and 3). The effects of all combination treatments on apoptosis were confirmed by assessment of nuclear morphology in all cell lines (Supplementary Figures 4 and 5).

### The effects of combination treatment on caspase 8 and 9 activity

**Figure 4:**
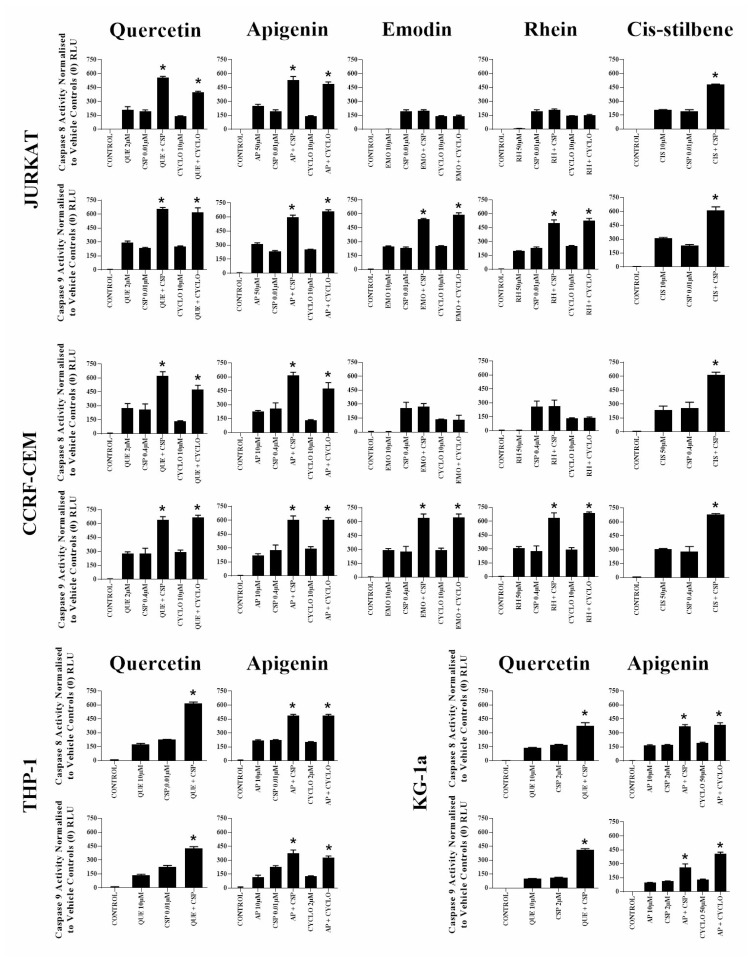
The effect of two alkylating agents: cisplatin (CSP) and cyclophosphamide (CYCLO) on caspases 8 and 9 activity when used in combination with quercetin (QUE), apigenin (AP), emodin (EMO), rhein (RH) or cis-stilbene (CIS) in lymphoid leukaemia cell lines (Jurkat and CCRF-CEM); and when used in combination with QUE or AP in myeloid leukaemia cell lines (THP-1 and KG-1a). This was evaluated by Caspases-Glo^®^ luminescent 8 and 9 assays. Cells were treated with CSP or CYCLO and polyphenols alone and in combination for 24h using their lowest-significant doses (LSD). Data were normalised to the vehicle control, which was assigned a zero luminescence (RLU). The data was expressed as medians with ranges (*N* = 4). Effects of combination treatments were statistically classified as synergistic (*) causing an increase in apoptosis or antagonistic (#) causing a decrease in apoptosis; when compared to vehicle control, drugs alone and expected values of combination treatments. Statistical significant was set at *P* ≤ 0.05.

When quercetin or apigenin were combined with cisplatin or cyclophosphamide, both caspase 8 and 9 activity were synergistically increased in all investigated leukaemia cell lines, consistent with caspase 3 and nuclear morphological assessments shown previously (*P* ≤ 0.05) (Figure 4). In contrast, emodin and rhein when used with cisplatin or cyclophosphamide only increased in caspase 9 activity in lymphoid leukaemia cells (*P* ≤ 0.05) (Figure 4); whereas *cis*-stilbene combined with cisplatin increased in caspase 8 and 9 activity, but only in lymphoid leukaemia cell lines (*P* ≤ 0.05) (Figure 4).

### The effect of combination treatments on cell cycle progression

In the lymphoid leukaemia cell lines, quercetin and apigenin were shown to have an interactive effect when combined with each alkylating agent, causing the accumulation of cells within S phase of cell cycle (*P* ≤ 0.05) (Supplementary Figures 6, 7 and Table 2). In myeloid cell lines, quercetin and apigenin treatment in combination with alkylating agents resulted in accumulation of cells in G_2_/M and/or S phase of the cell cycle (*P* ≤ 0.05) (Supplementary Figures 6 and 7, Table 2). The only exception was when quercetin and cyclophosphamide were combined in myeloid cell lines, where an antagonistic effect was observed with no cell cycle arrest (*P* ≤ 0.05) (Supplementary Figure 6 and Table 2).

**Table 2:** The effect of cisplatin (CSP), cyclophosphamide (CYCLO) and chlorambucil (CLB) on cell cycle progression, when used in combination with quercetin (QUE), apigenin (AP), emodin (EMO), rhein (RH), or cis-stilbene (CIS) in two lymphoid leukaemia cell lines (Jurkat and CCRF-CEM) and two myeloid leukaemia cell lines (THP-1 and KG-1a)

	Lymphoid Leukaemia						Myeloid Leukaemia					
	Jurkat			CCRF-CEM			THP-1			KG1a		
	CSP	CYCLO	CLB	CSP	CYCLO	CLB	CSP	CYCLO	CLB	CSP	CYCLO	CLB
Quercetin	SYN Interactive S	SYN InteractiveS	ADD Non-Interactive S	SYN Interactive S	SYN Interactive S	SYN Interactive S	SYN Interactive G2/M	ANTG No Arrest	SYN Interactive G2/M	SYN Interactive G2/M	ANTG No Arrest	SYN Interactive S
Apigenin	SYN Interactive S	SYN Interactive S	SYN Interactive S	SYN Interactive S	SYN Interactive S	SYN Interactive S	SYN Interactive G2/M	SYN Interactive S & G2/M	SYN Interactive S & G2/M	SYN Interactive G2/M	SYN Interactive G0/ G1	SYN Interactive G2/M
Emodin	SYN Interactive S	SYN Interactive G0/ G1	ADD Non-Interactive G0/ G1	SYN Interactive S	SYN Interactive S	SYN Interactive S	ANTG No Arrest	ANTG No Arrest	SYN Interactive G2/M	ANTG No Arrest	ANTG No Arrest	SYN Interactive S & G2/M
Rhein	SYN Interactive S	SYN Interactive G0/ G1	SYN Interactive G0/ G1	SYN Interactive S	SYN Interactive S	SYN Interactive S	ANTG No Arrest	ANTG No Arrest	ANTG No Arrest	ANTG No Arrest	ANTG No Arrest	ANTG No Arrest
Cis-Stilbene	SYN Interactive S	ANTG No Arrest	ANTG No Arrest	SYN Interactive S	ANTG No Arrest	ANTG No Arrest	ANTG No Arrest	ANTG No Arrest	ANTG No Arrest	ANTG No Arrest	ANTG No Arrest	ANTG No Arrest

Cell cycle progression was analysed by flow cytometry following propidium iodide staining. Cells were treated with CSP, CYCLO or CLB and polyphenols alone and in combination for 24 h using their lowest-significant doses (LSD) as determined by CellTiter-Glo assay, together with a vehicle control. The percentage of cells in each phase was analysed with FlowJo software using the Watson (Pragmatic) model. The data were expressed as medians with ranges (*N =* 4). Statistical significance of combination treatments was determined and compared with the vehicle control and the individual treatments alone. Statistical significance was set at *P* ≤ 0.05. The combination effects of drugs were statistically shown to be either: non-interactive (additive effect), interactive (synergistic (SYN) effect) or no arrest (antagonistic (ANTG) effect) on the cell cycle phases, this has been statistically determined as shown in our previous work^17^.

When emodin and rhein were used in combination with each alkylating agent, an interactive effect was observed, with an accumulation of cells in G_0_/G_1_ or S phase of the cell cycle in lymphoid cell lines (*P* ≤ 0.05) (Supplementary Figures 8, 9 and Table 2), whilst in the myeloid cell lines, both emodin and rhein antagonised the alkylating agent-induced cell cycle arrest (*P* ≤ 0.05) (Table 2). However, when emodin was used in combination with chlorambucil in myeloid cell lines, an interactive effect was observed with S and/or G_2_/M phase cell cycle arrest (*P* ≤ 0.05) (Table 2).

In contrast, *cis*-stilbene when used with each alkylating agent had no effect on cell cycle in either the lymphoid or myeloid cell lines (*P* ≤ 0.05) (Supplementary Figure 10 and Table 2). The only exception was seen when *cis*-stilbene was used in combination with cisplatin in lymphoid cell lines, where there was S phase cell cycle arrest (*P* ≤ 0.05) (Table 2).

### The effect of combination treatments on glutathione levels

**Figure 5:**
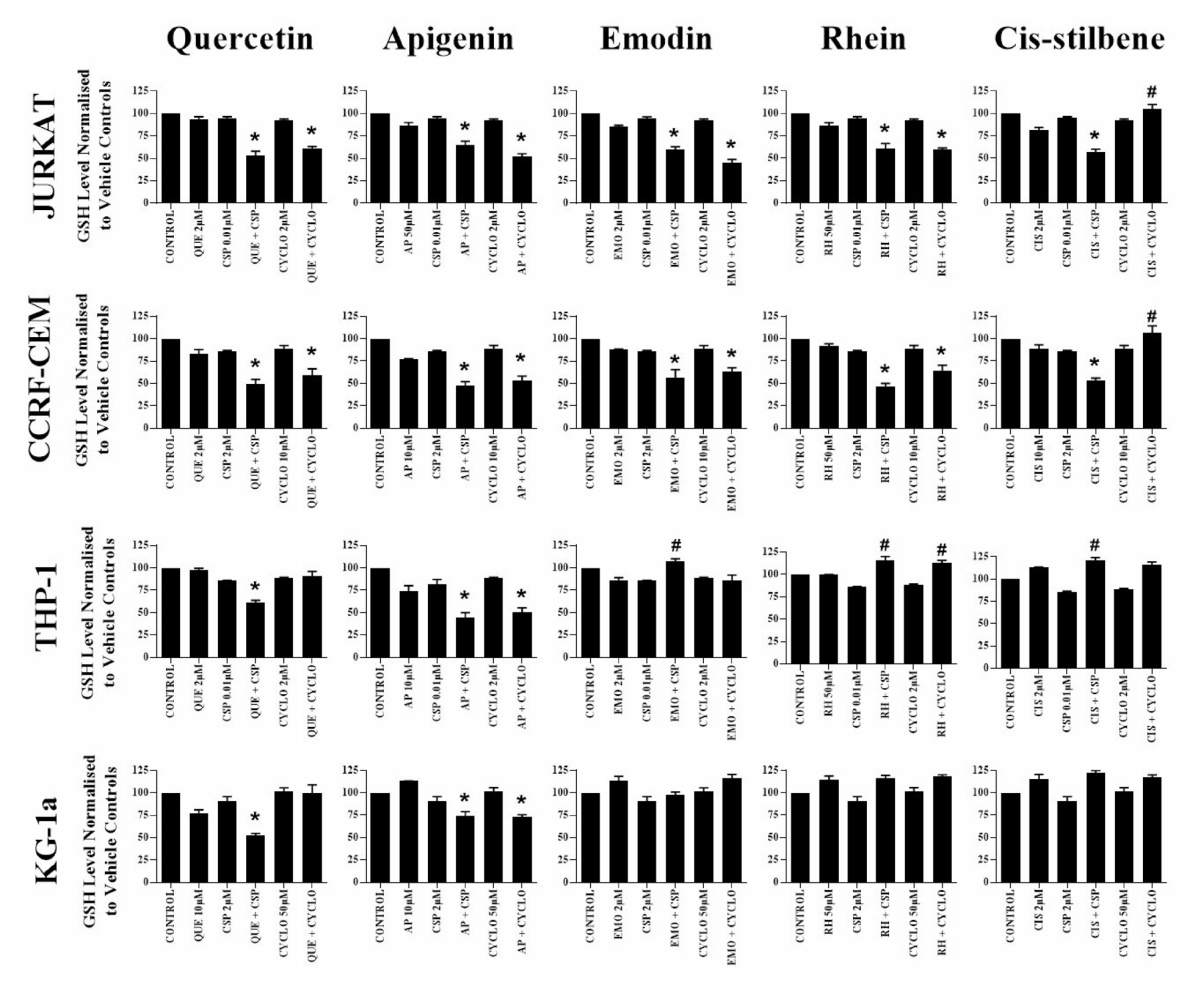
The effect of two alkylating agents: cisplatin (CSP) and cyclophosphamide (CYCLO) on glutathione (GSH) levels when used in combination with quercetin (QUE), apigenin (AP), emodin (EMO), rhein (RH) or cis-stilbene (CIS) in two lymphoid (Jurkat and CCRF-CEM); and two myeloid leukaemia cell lines (THP-1 and KG-1a). GSH levels were evaluated by the GSH-Glo™ Glutathione. Cells were treated with CSP or CYCLO and polyphenols alone and in combination for 24 h using their lowest-significant doses (LSD). Data was normalised to the vehicle control which was assigned 100% of GSH levels. The data was expressed as medians and ranges (*n* = 4). Effects of combination treatments were statistically classified as synergistic (*) causing a decrease in GSH levels or antagonistic (#) causing an increase in GSH levels; when compared to vehicle control, drugs alone and expected values of combination treatments. Statistical significant was set at *P* ≤ 0.05.

Glutathione analysis was performed for alkylation agents and polyphenol combinations which previously showed synergistic effects across both lymphoid and myeloid leukaemia cell lines (Figure 5). Following apigenin combination treatments with cisplatin or cyclophosphamide, glutathione levels were synergistically reduced in all leukaemia cell lines (*P* ≤ 0.05) (Figure 5). Similarly a decrease in glutathione levels were also observed when quercetin was combined with cisplatin in all leukaemia cell lines (*P* ≤ 0.05) (Figure 5), and with cyclophosphamide in lymphoid cell lines only (*P* ≤ 0.05) (Figure 5).

Emodin and rhein were also shown to synergistically reduce glutathione when used in combination with cisplatin and cyclophosphamide in lymphoid cell lines (*P* ≤ 0.05) (Figure 5). However, this effect was reversed in THP-1 myeloid cells, with emodin and rhein antagonising the action of cisplatin and cyclophosphamide, causing a significant increase in glutathione levels (*P* ≤ 0.05) (Figure 5). In contrast, *cis-*stilbene was shown to synergize the action of cisplatin in lymphoid cell lines and reduce levels of glutathione; but antagonise the action of cisplatin and cyclophosphamide in THP-1 myeloid cell lines and cyclophosphamide in all the lymphoid cell lines; and significantly elevating the levels of glutathione (*P* ≤ 0.05) (Figure 5). CMFDA staining confirmed these results (Supplementary Figure 11), showing similar responses observed using the GSH-Glo™ glutathione assay.

### The effect of combination treatments on γH2AX foci

**Figure 6:**
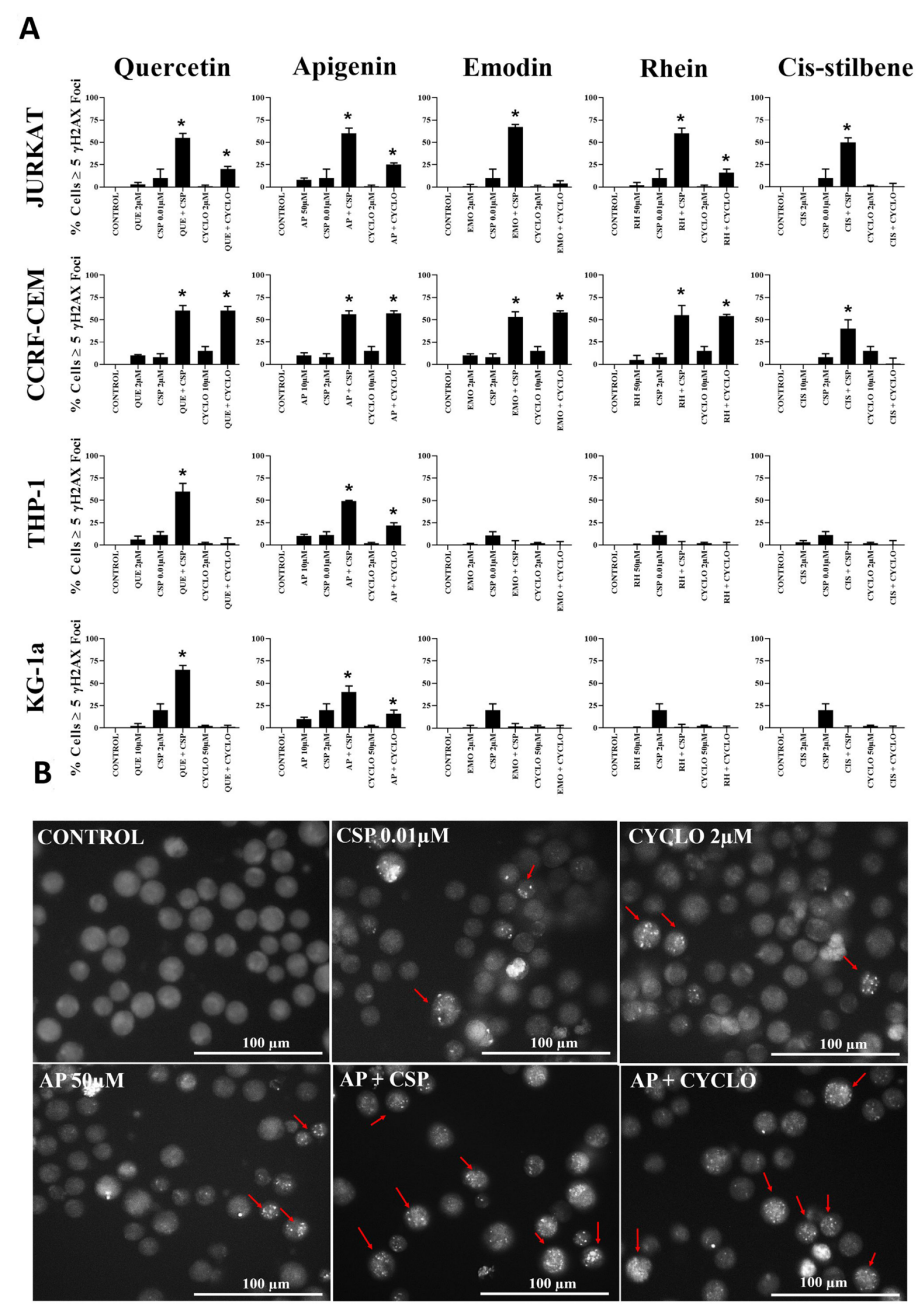
(**A**) The effect of two alkylating agents: cisplatin (CSP) and cyclophosphamide (CYCLO) on γ-H2AX foci formation (DNA damage marker) when used in combination with quercetin (QUE), apigenin (AP), emodin (EMO), rhein (RH) or cis-stilbene (CIS); in two lymphoid (Jurkat and CCRF-CEM) and two myeloid (THP-1 and KG-1a) leukaemia cell lines. This was evaluated by the immunofluorescent staining using Alexa Fluor^®^ 647 Mouse anti-H2AX (pS139). Cells were treated with CSP, CYCLO or CLB and polyphenols alone and in combination for 24 h using their lowest-significant doses (LSD). The data was expressed as medians and ranges (*N =* 4). Data was normalised to the vehicle control which was assigned 0% of γ-H2AX foci formation (DNA damage marker). Effects of combination treatments were statistically classified as synergistic (*) causing an increase in the percentage of cells with γ-H2AX foci or antagonistic (#) causing a decrease in the percentage of cells with γ-H2AX foci; when compared to vehicle control, drugs alone and expected values of combination treatments. Statistical significant was set at *P* ≤ 0.05. (**B**) An example of immunofluorescent detection of DNA damage (measured as γ-H2AX foci) for Jurkat lymphoid leukaemia cells when treated with LSDs of cisplatin (CSP) and cyclophosphamide (CYCLO) alone and in combination with apigenin (AP) for 24 h. Cells with ≥5 foci (red arrows) are identified as having DNA damage. Images were captured in bright filed using (Cell-F software, Olympus). Scale bar = 100 μm.

DNA damage analysis was undertaken in those combination treatments which showed the most promising synergistic effect in all leukaemia cell lines (Figure 6). Cisplatin and cyclophosphamide alone caused an increase in levels of DNA damage in all leukaemia cell lines (*P* ≤ 0.05) (Figure 6). Following apigenin combination treatment with cisplatin or cyclophosphamide a synergistic increase in γH2AX foci was seen in all leukaemia cell lines (*P* ≤ 0.05), shown here in Jurkat cells (Figure 6B). Likewise, quercetin synergistically increased γH2AX foci when combined with cisplatin in all leukaemia cell lines (*P* ≤ 0.05) and when combined with cyclophosphamide, in the lymphoid cell lines (*P* ≤ 0.05) (Figure 6).

Emodin and rhein were also shown to synergize the formation of γH2AX foci when used in combination with both cisplatin and cyclophosphamide, but only in lymphoid cell lines (*P* ≤ 0.05), with no significant effects in myeloid cell lines (Figure 6). In contrast *cis-*stilbene synergistically increased γH2AX foci in combination with cisplatin in lymphoid cell lines (*P* ≤ 0.05); but not myeloid cell lines (Figure 6).

## DISCUSSION

This study investigated the combined effects of five polyphenols (quercetin, apigenin, emodin, rhein, and cis-stilbene) on three alkylating agents (cisplatin, cyclophosphamide and chlorambucil). Our earlier work has shown that these polyphenols can induce apoptosis and arrest cell cycle in leukaemia cell lines [11]. Here, we show the effects of combination treatments on ATP levels, the induction of apoptosis and cell cycle arrest in non-tumour control cells and leukaemia cell lines. In non-tumour control cells, all polyphenols were shown to inhibit the toxic effects of all alkylating agents, suggesting that polyphenols are protective of normal cells.

In lymphoid leukaemia cells, quercetin, apigenin, emodin and rhein synergistically enhanced cisplatin and cyclophosphamide activity, reduced ATP levels, causing cell cycle arrest and inducing apoptosis. Similarly, chlorambucil acted synergistically with apigenin or rhein. However, in myeloid leukaemia cells all three alkylating agents displayed differential effects, some synergistic and some antagonistic, when combined with polyphenols.

Interestingly, the most promising polyphenols for the combination with all alkylating agents in all leukaemia cell lines, were apigenin and quercetin; and the least effective polyphenol was *cis*-stilbene. This is a novel finding, and to date no previous studies have combined cyclophosphamide and chlorambucil with our selected polyphenols. There is however, previous work which combined cisplatin with apigenin, quercetin and emodin in solid tumours [25–27]. Apigenin has been shown to enhance the action of cisplatin in head and neck squamous cells (SCC25) [25], human nasopharyngeal (CNE-2Z) [26] and laryngeal carcinoma (Hep-2) cell lines [27]. A similar effect was observed here, with apigenin enhancing the action of all three alkylating agents in all leukaemia cell lines, synergistically reducing ATP and glutathione levels, inducing DNA damage and cell cycle arrest.

Similarly, quercetin has previously been shown to enhance the activity of cisplatin, and synergistically induce apoptosis in a number of solid tumours [18–24]. Furthermore, the combination of cisplatin and quercetin was also been shown to induce synergistic arrest of the cell cycle at S phase in human malignant mesothelioma cells (SPC212 and SPC111) [21], and at G_0_/G_1_ phase, by elevation of p16^INK4A^ expression in human hepatocellular carcinoma cells (HepG2) [24].

Cisplatin has been previously shown to be potentiated by other polyphenols such as emodin, in non-small cell lung cancer (NSCLC) with HER-2/neu-overexpressing [28], merkel cell carcinoma (MCC) [29], human ovarian tumour cell lines (A2780) [30], and gallbladder carcinoma cells (SGC996) [31, 32]. Likewise, here cisplatin and cyclophosphamide in combination with quercetin synergistically reduced ATP levels, induced apoptosis, and accumulated the cells at S phase and/or G_2_/M phase in both lymphoid and myeloid cell lines. The only exception was seen in myeloid cell lines where an antagonistic effect was seen when cyclophosphamide was combined with quercetin.

This current study showed that emodin and rhein in combination with alkylating agents caused a synergistic reduction in ATP levels, induction of apoptosis and accumulation of cells at S phase in lymphoid leukaemia cell lines; whilst having a differential effects in myeloid leukaemia cell lines, some of which were antagonistic. Demonstrating the therapeutic effects of alkylating agents in combination emodin and rhein may be dependent on the cell lineage; and could be useful in the treatment of lymphoid leukaemia, but may be detrimental in myeloid leukaemia.

C*is*-stilbene was the least effective polyphenol producing mostly antagonistic effects when used in combination with cyclophosphamide and chlorambucil in both lymphoid and myeloid leukaemia cells, the only exception was seen when used in combination with cisplatin which produced synergistic effects in lymphoid cells.

In the current study, all the synergistic responses of polyphenols with either cisplatin or cyclophosphamide were accompanied by a down-regulation of glutathione and increased DNA damage. This resulted in a synergistic increase in caspase 8 and 9 when combined with quercetin or apigenin, and caspase 9 when combined with emodin or rhein in each investigated leukaemia cell line. This ultimately led to a synergistically increased in caspase 3 activity and apoptotic cell death.

These results suggest that synergistic effects of alkylating agents with quercetin or apigenin in both myeloid and lymphoid cell lines is dependent on activation of both extrinsic and intrinsic apoptotic pathways; whilst their effects with emodin or rhein are dependent on caspase 9, and the activation of the intrinsic apoptosis pathways. However, as these cells are p53 null [33–35], this could not occur via the p53 pathway; suggesting that their action is via p53 independent Bad and/or Bak activity. Previous studies have shown similar effects with cisplatin when combined with quercetin, causing a synergistic induction of the intrinsic apoptotic pathway, an increase in caspase 9 and 3 activity. This was shown to occur via a down-regulation of Bcl-xl and Bcl-2, and an increase in cytochrome-C in a number of solid tumour cells [19–21]. Furthermore, synergistic treatments in human non-small cell lung carcinoma (NCL-H-520) cell lines demonstrated interactive effects on cell cycle resulting in S phase and/or G_2_/M phase arrest; accompanied by the presence of γH2AX foci, which are indicative of DNA damage [19].

Current studies report that the measurement of γH2AX foci are useful in determining whether or not drugs reach a tumour, are convert to their activated forms, and are capable of inducing DNA damage [36, 37]. Rajendran *et al*, (2011) suggested that the use of combination treatments which cause DNA damage and interfere with DNA repair in cancer cells could be very effective and produce synergistic effects [38]. In this regard, the synergistic drug combinations reported here are worthy of further investigation in leukaemia.

In contrast, the antagonistic responses seen here when cisplatin and cyclophosphamide were combined with emodin, rhein or cis-stilbene in myeloid cell lines were associated with elevation in glutathione levels, and no γ-H2AX foci and hence no DNA damage, or cell cycle arrest. There is limited evidence that shows polyphenols can block the activity of standard chemotherapy. However, Cˇipak *et al* 2003, showed apigenin, galangin and chrysin inhibited the action of cisplatin and doxorubicin, in murine leukaemia (L1210) cells [39]. This antagonistic effect has been attributed to the polyphenols’ antioxidant activity, which protecting the cells against cisplatin and doxorubicin generated reactive oxygen species (ROS). It was suggested that apoptosis was inhibited, by suppression of ROS production and the activation of the c-Jun NH2-terminal kinase (JNK) pathway [39]. Likewise, it was reported that rutin hydrate, quercetin dehydrate, hydrocaffeic acid, gallic acid and tannic acid antagonised the induction of apoptosis caused by bortezomib in multiple myeloma cells (MC/CAR, RPMI8226 and U266) [40]. However, the exact mechanism action of these polyphenols on bortezomib remains unknown.

Earlier work has also reported polyphenols can act as both an antioxidants and pro-oxidants, depending on doses and cellular conditions. This suggests that polyphenols are capable of modulating antioxidant redox systems, such as glutathione [41–43], and thus could have a chemopreventive effect. Alternatively, the anti-cancerous effects of polyphenols could be attributed to their pro-oxidant activity [41–43].

Here we have shown the synergism and antagonism mechanisms of action of our selected polyphenols with cisplatin and cyclophosphamide, are strongly dependent on modulation of glutathione levels, caspase activity and DNA damage within each studies cell lines. These findings are supported by earlier studies, where cisplatin when combined with quercetin [44], apigenin [25] or emodin [31] synergistic inhibited cell proliferation and induced apoptosis through the elevation of intracellular ROS and the reduction glutathione levels. This caused mitochondrial transmembrane potential dissipation and activated of the intrinsic apoptotic pathway via release of cytochrome-C, down-regulation of Bcl-X(L) and up-regulation Bax, followed by caspase 9 and 3 activation and apoptosis in ovarian [44], head and neck squamous carcinoma (SCC25) [25] and gallbladder (SGC996) [31] cancer cell lines.

Here, lymphoid leukaemia cell lines demonstrated greater synergistic responses to the combination of alkylating agents and polyphenols than myeloid leukaemia cell lines. Indeed, our previous work has shown that in leukaemia cell lines, basal glutathione levels correlate strongly with responses to combination treatments of topoisomerase inhibitors and polyphenols [17]. In particular, we previously reported that low basal glutathione levels were present in lymphoid leukaemia cell lines compared to non-tumour control cells and myeloid cell lines; and lymphoid leukaemia cell lines were more sensitive to this combination treatment [17], whereas higher basal glutathione levels in myeloid leukaemia cells and non-tumour controls cells was associated with resistance to combination treatments of polyphenols and topoisomerase inhibitors [17]. This may explain why lymphoid cell lines here were more sensitive to alkylating agents/polyphenol combination treatments than the myeloid leukaemia cell lines. Traverso *et al*, 2013 reported that cancers with high levels of glutathione such as lung, ovarian, breast, and haematological malignancies have an increase in the antioxidant capacity and this prevented oxidative stress and DNA damage, and inhibited apoptosis; and as a result, this could also lead to drug-resistance [42]. Furthermore, it is reported that cancers with low levels of glutathione such as melanoma have a decrease of cellular antioxidant capacity and an increase in oxidative stress, DNA damage and cell death; and this can lead to increases in the sensitivity of these cancers to treatment [42]. Thus, cancer cell types and basal glutathione levels may be relevant when designing new clinical chemotherapy regimens. It is apparent that polyphenols have the capacity to reduce basal glutathione levels in cancer cells and increase susceptibility to chemotherapy treatment.

Additionally, our previous work reported that glutathione depletion helped to increase the therapeutic efficacy of topoisomerase inhibitors [17, 45]. It is believed that the reduction of glutathione can predispose cells to apoptosis, directly trigger the formation of permeability transition pore, and the activation of execution caspases [46]. Furthermore the over expression of anti-apoptotic Bcl-2, and the inhibition of mitochondrial-induced apoptosis, are strongly linked to the antioxidant property of glutathione [46]. Therefore, it is necessary to decrease glutathione levels, in order to increase the efficacy of chemotherapy agents and reduce multidrug resistance in cancer cells [47–49].

In conclusion, this study suggests that the combination of alkylating agents, in particular cisplatin with polyphenols show promising anti-tumour activities in lymphoid leukaemias. However, the potential antagonistic effects of some polyphenols on certain alkylating agents need careful consideration and further investigation.

## MATERIALS AND METHODS

### Leukaemia cell lines

Four human leukaemia cell lines were used for this study: Two lymphoid leukaemia cell lines (Jurkat (peripheral blood T cell leukaemia) (ATCC: TIB-152, Middlesex, UK) and CCRF-CEM (acute lymphoblastic leukaemia) (ATCC: CCL-119, Middlesex, UK)); which had been previously shown to be sensitivity to polyphenols treatments [17]; together with two myeloid leukaemia cell lines (THP-1 (acute monocytic leukaemia) (ATCC: TIB- 202, Middlesex, UK) and KG-1a (acute myelogenous leukaemia) (ATCC:CCL-243)) which had been previously shown to be resistance to polyphenol treatment [17]. Non-tumour control hematopoietic stem cells from cord blood (CD34+HSC and CD133+HSC) (Stem cell Technologies, Grenoble, France) were also included in the study. All cells were tested for mycoplasma contamination using the MycoAlert TM mycoplasma detection kit (Lonza, MD, USA) and were negative throughout the study.

### Culture conditions

Cells were maintained in Roswell Park Memorial Institute (RPMI) medium 1640 (Invitrogen, Paisley, UK) supplemented with 10% (v/v) foetal bovine serum, 1.5 mM L-glutamine and 100 μg/ml penicillin/streptomycin at 37º C with 5% CO2.

### Treatment regimes

Two lymphoid leukaemia cell lines (Jurkat and CCRF-CEM), two myeloid leukaemia cell lines (THP-1 and KG-1a); and two non-tumour control cells (CD34+HSC and CD133+HSC) were treated with each polyphenol and each alkylating agent: cisplatin (CSP), cyclophosphamide (CYCLO), chlorambucil (CLB) (Sigma, Poole, UK) alone or in combination, along with a vehicle control for 24 h. All treatments were performed in triplicate with four technical replicated.

Quercetin (QUE) (Enzo Life Sciences, Exeter, UK), apigenin (AP), emodin (EMO), rhein (RH) and *cis*-stilbene (CIS) (Sigma, Poole, UK) were prepared as described previously [11]. Cisplatin was dissolved in 1:1 v/v water: ethanol; and cyclophosphamide and chlorambucil were dissolved in water. Stock solutions (25 mM) of each polyphenol were prepared with 10% (v/v) ethanol (Sigma) in serum free media (Invitrogen, Paisley, UK) to generate treatment concentrations between 0.005 and 50 μM. All polyphenols were HPLC analytical grade and were 90-97% pure. In addition, 0.1% (v/v) ethanol vehicle controls were prepared for each treatment in all cell lines.

Dose response curves were generated for each alkylating agent. These were used to determine the IC50 dose (which statistically reduced ATP levels by 50%) and lowest significant dose (LSD) (which is the lowest dose which statistically reduced ATP levels and induced caspase 3 activity when compared to the vehicle control at 24 h) (Table 1). Significance was determined using a Kruskal-Wallis with a Conover-Inman Post hoc-test. The LSDs for each polyphenol were determined in our previous study [17]. These LSD doses were used for subsequent polyphenol/ chemotherapy combination treatments.

Cells were treated with each polyphenol and each alkylating agent: cisplatin (CSP), cyclophosphamide (CYCLO), chlorambucil (CLB) alone or in combination at their LSDs, along with a vehicle control, for 24 h. All treatments were performed in triplicate with four technical replicates. Following treatments with each alkylating agent, ATP levels, cell cycle progression, induction of apoptosis and caspase 3 activity were assessed. Subsequently, assessment was made of glutathione levels and DNA damage (measured as γH2AX foci) following cisplatin and cyclophosphamide treatments.

### CellTiter-Glo^®^ luminescent cell ATP viability assay

The CellTiter-Glo^®^ luminescent assay measure ATP levels and is indicative of cell viability and proliferation. Cells were seeded at 2.5 × 10^3^ cells per well of white 96-well plates (Fisher Scientific, Loughborough, UK) and treated with each polyphenols and alkylating agents alone and in combination for 24 h, together with a 0.1% (v/v) ethanol vehicle control. Following treatments, the CellTiter-Glo^®^ luminescent cell viability assay (Promega) was used to measure ATP levels, as per manufacturer’s instructions. Dose response curves were generated for each alkylating agent. These were used to determine the lowest significant dose (LSD) which statistically reduced ATP levels when compared to the vehicle control at 24 h (Table 1). These LSD for ATP were used for subsequent polyphenol/chemotherapy combination work on cell viability, cell-cycle progression, DNA damage and glutathione levels.

### Apoptosis and cell cycle analysis

Cells were seeded at 0.5 × 10^6^ cells per well in 12-well plates and treated with polyphenols and alkylating agents alone or in combination for 24 h, together with a 0.1% (v/v) ethanol vehicle control. Following treatments, caspase 3 activity was determined using Nucview caspase 3 activity assay using flow cytometry, as described previously [17]. Dose response curves were generated for each alkylating agent. These were used to determine the lowest significant dose (LSD) which statistically increased caspase 3 activity compared to the vehicle control at 24 h (Table 1). These LSD doses for caspase 3 activity were used for subsequent polyphenol/chemotherapy combination treatments on the morphological assessment of apoptosis using Hoechst 33342/propidium iodide staining and fluorescence microscopy; and for the investigation of cell cycle progression using propidium iodide staining and flow cytometry, as described previously [17].

### Caspases 8 and 9-Glo^®^ luminescence assays

Cells were seeded into white 96-well plates (Fisher Scientific, Loughborough, UK) at 2.5 × 10^3^ cells per well and treated with two alkylating agents: cisplatin and cyclophosphamide. These alkylating agents were selected as they showed a greater synergistic effect when used in combination with polyphenols; across both lymphoid and, myeloid leukaemia cell lines. These alkylating agents were used alone and in combination with those polyphenols that previously showed a synergistic effect and were used at their LSDs for 24 h, together with a 0.1% (v/v) ethanol vehicle control. Following treatment Caspase-Glo^®^ 8 and 9 assays were used as per manufacturerʼs instructions (Promega, Southampton, UK) to determine caspase 8 and 9 activity. Luminescence was measured using a Wallac Victor 2 1420 and were normalised to vehicle controls.

### Measurement of cell glutathione (GSH) levels GSH-Glo™ glutathione assay

Cells were seeded at 2.5 × 10^3^ cells per well into white 96-well plates and treated with two alkylating agents (cisplatin and cyclophosphamide) alone or in combination with polyphenols at their LSD for 24 h, together with vehicle control. Following treatment, glutathione levels were measured using the GSH-Glo^TM^ glutathione luminescent assay as per the manufacturer’s instructions and reported previously [17]. The luminescent signal was measured using a Wallac Victor 2 1420 and normalised to the vehicle control.

### Cell Tracker™ Green 5-Chloromethylfluorescein Diacetate (CMFDA)-glutathione staining

Cells were seeded into white 24-well plates (Fisher Scientific, Loughborough, UK) at 1.0 × 10^3^ cells per well and treated with two alkylating agents (cisplatin and cyclophosphamide) and polyphenols alone and in combination at their LSD for 24 h, together with a vehicle control; then incubated at 37° C with 5% CO_2_ for 24 h. Following treatments, cells were stained with Cell Tracker™ Green CMFDA (Molecular Probes) to detect and quantify GSH levels (specificity 95%) and counterstained with Hoechst 33342 stain (Sigma, Poole UK) to localize the cell nuclei as described previously [17]. Images were captured using Cell^F^ imaging software (Olympus). CMFDA green staining was localised mainly in the cytoplasm, as well in the nucleus of the live cells only, which commonly have high glutathione level, while it is depleted in apoptotic and dead cells. Hoechst 33342 blue staining was commonly localised in the nuclei of both live and dead cells. Two hundred cells (green and blue) were counted and then percentage of (green stained) GSH positive cells was determined for each sample.

### Measurement of DNA damage by γH2AX foci detection

The formation of γH2AX foci is indicative of DNA damage. The Alexa Fluor 647 Mouse anti-γH2AX (pS139) (BD Pharmingen, Oxford, UK) specifically targets phosphorylation of Ser-139 at the C-terminal region of γH2AX enabling the visualisation of γH2AX by immunofluorescence. Cells were seeded at 1.0 × 10^3^ cells per well into a BD Falcon 96-well imaging plate (BD Pharmingen) and treated with two alkylating agents: cisplatin and cyclophosphamide) alone and in combination with each polyphenol at their LSD for 24 h, together with vehicle control. Following treatments, cells were centrifuged at 400 × g for 10 min then washed in PBS and fixed in BD Cytofix fixation buffer for 10 min (BD Pharmingen). Cells were washed twice in PBS and permeabilized in 90% methanol (Sigma) for 5 min. Following washes, cells were incubated in 50 μl Alexa Fluor 647 Mouse anti-γH2AX (pS139) (1:10 v/v) at room temperature for 1 h in the dark. Cells were washed 3 times in PBS and counter-stained in 100μl of 2 mg/ml Hoechst 33342 stain for 15 min.

Cells were visualised using inverted fluorescence microscopy (Olympus IX-81) and analysed using Cell-F software (Olympus). Cells with greater than six γ-H2AX foci were considered as having DNA damage. The number of cells with DNA damage (>6 foci) or without DNA damage (<6 foci) were counted. Two hundred cells per treatment were counted and percentage of cells with substantial DNA damage determined. Images were captured using an inverted fluorescence microscopy and the Cell-F software*.*

### Statistical analysis

The median with range was calculated for each assay. Stats Direct software (Stats Direct Ltd, Altrincham, UK) was used to test whether data followed a normal distribution using normality testing in Stats Direct. As data was non-parametric, a Kruskal-Wallis and Conover-Inman post-hoc test was used to determine the statistical significance of the data. Results were considered statistically significant when *P* ≤ 0.05.

The effect of combination chemotherapy and polyphenol treatment on ATP levels, apoptosis, glutathione levels and DNA damage were classified as: synergistic (^*^) or antagonistic (#) as described previously [17]. All results were considered statistically significant when *P* ≤ 0.05.

Cell-cycle analysis was performed by determining the percentage of cells in each phase using the FlowJo software using the Watson pragmatic model. The data was expressed as medians with ranges (*N =* 4). The statistical significance of individual drugs was determined firstly in comparison to the vehicle control using a Kruskal-Wallis and Conover Inman post-hoc tests. The statistical significance of combined polyphenol and drugs treatments was determined in comparison to the vehicle control and the individual treatments alone. The effect of combination treatments on cell-cycle was classified either as: interactive (synergetic), non-interactive or no arrest (antagonistic) using the method described previously [17]. Statistical significance was set at *P* ≤ 0.05.

## SUPPLEMENTARY MATERIALS



## Abbreviations

CSP: Cisplatin
CYCLO: Cyclophosphamide
CLB: Chlorambucil
QUE: Quercetin
AP: Apigenin
EMO: Emodin
RH: Rhein
CIS: *Cis*-Stilbene
