# Supplementary material for: Polyphenols enhance the activity of alkylating agents in leukaemia cell lines
Source: Oncotarget. 2019 Jul 16;10(44):4570–86. doi: 10.18632/oncotarget.27068 (PMC6642044; doi:10.18632/oncotarget.27068)
Supplement: Supplementary file 1 [file oncotarget-10-4570-s001.pdf]

Polyphenols enhance the activity of alkylating agents in leukaemia cell lines

SUPPLEMENTARY MATERIALS

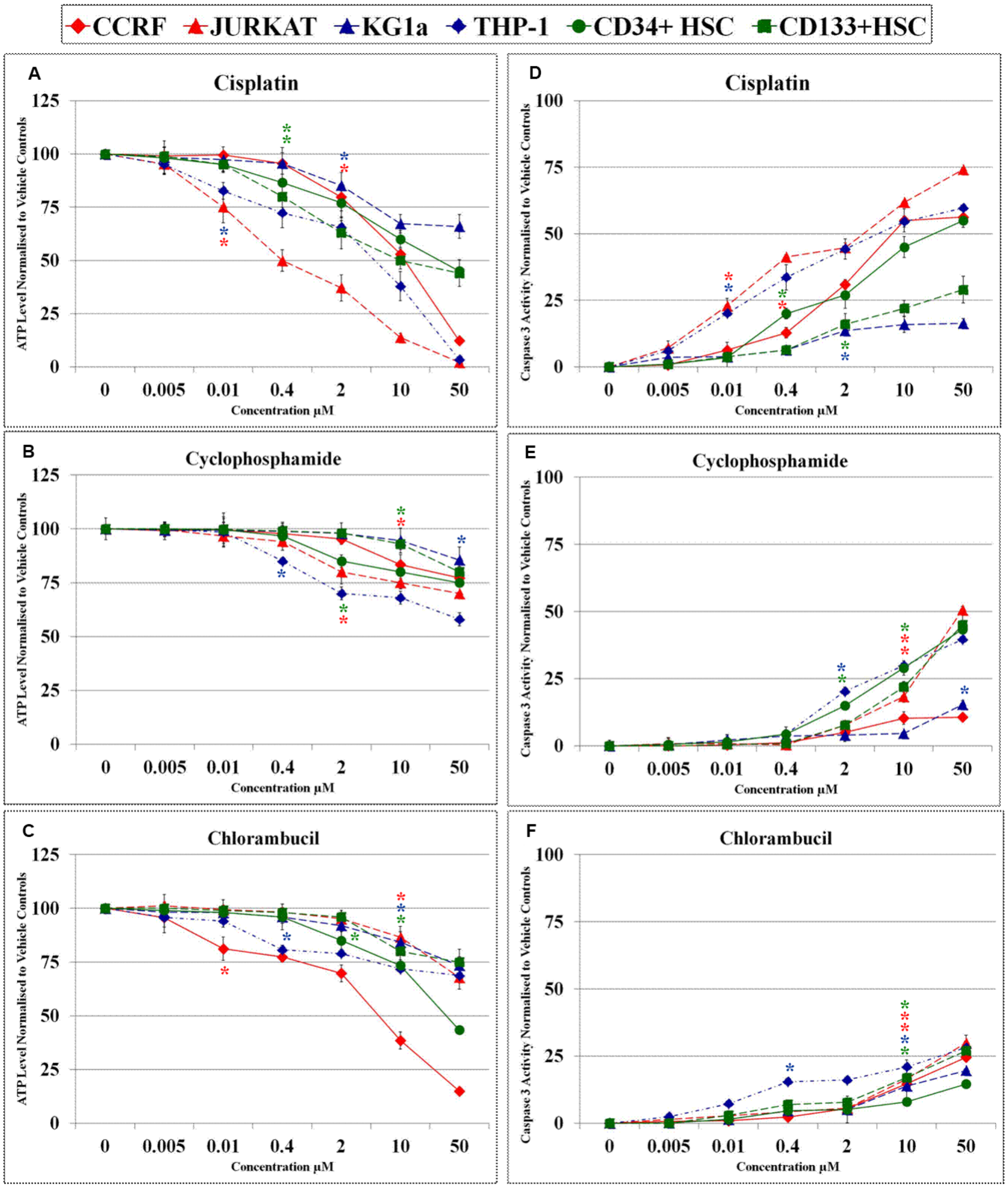

Supplementary Figure 1: Effect of cisplatin, cyclophosphamide and chlorambucil alone on ATP levels (**A**–**C**) and caspase 3 activity (**D**–**F**) in two lymphoid (CCRF-CEM and Jurkat), two myeloid (THP-1 and KG-1a) leukaemia cell lines, and two non-tumour control cells (CD133+HSC and CD34+ HSC). The lowest significant doses (LSD): which caused a significant reduction on ATP levels and induction of caspase 3 activity when compared to the vehicle control were determined for each alkylating agent in each cell lines. The *indicated for LSD in each cell line.

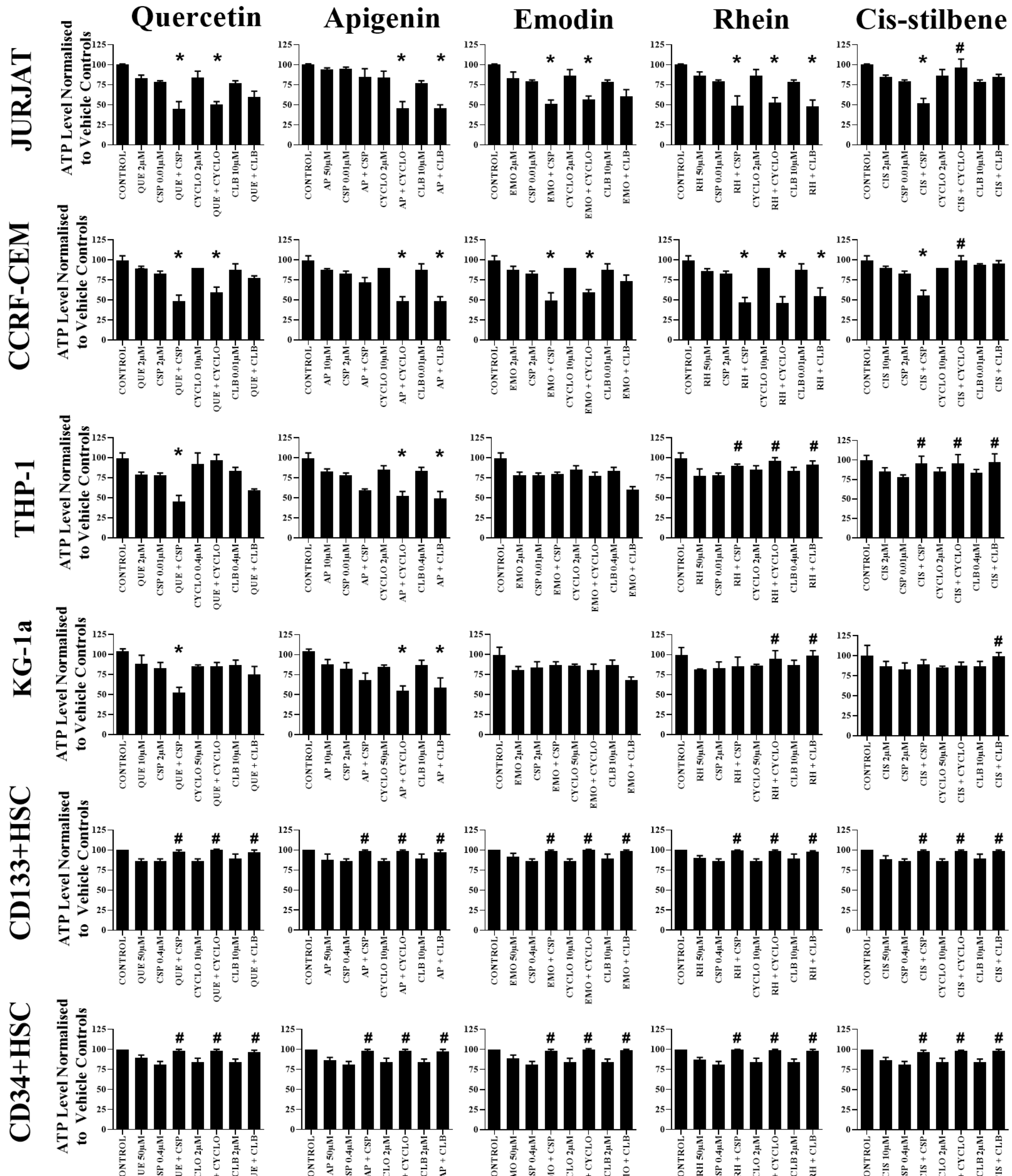

Supplementary Figure 2: The effect of cisplatin (CSP), cyclophosphamide (CYCLO) and chlorambucil (CLB) when used in combination with quercetin (QUE), apigenin (AP), emodin (EMO), rhein (RH), or cis-stilbene (CIS) on ATP levels; in two lymphoid (Jurkat and CCRF-CEM) and two myeloid (THP-1 and KG-1a) leukaemia cell lines, and two non-tumour control cells (CD133+ HSC and CD34+ HSC). This was evaluated by CellTiter-Glo® assay. Cells were treated with CSP, CYCLO or CLB and polyphenols alone and in combination for 24 h using their lowest-significant doses (LSD); together with a vehicle control. All data was normalised to the vehicle control which was assigned 100% cell viability. The data was expressed as medians and ranges ($N = 4$). Effects of combination treatments were statistically classified as synergistic (*) causing a decrease in ATP levels or antagonistic (#) causing an increase in ATP levels; when compared to vehicle control, drugs alone and expected values of combination treatments. Statistical significant was set at $P \leq 0.05$.

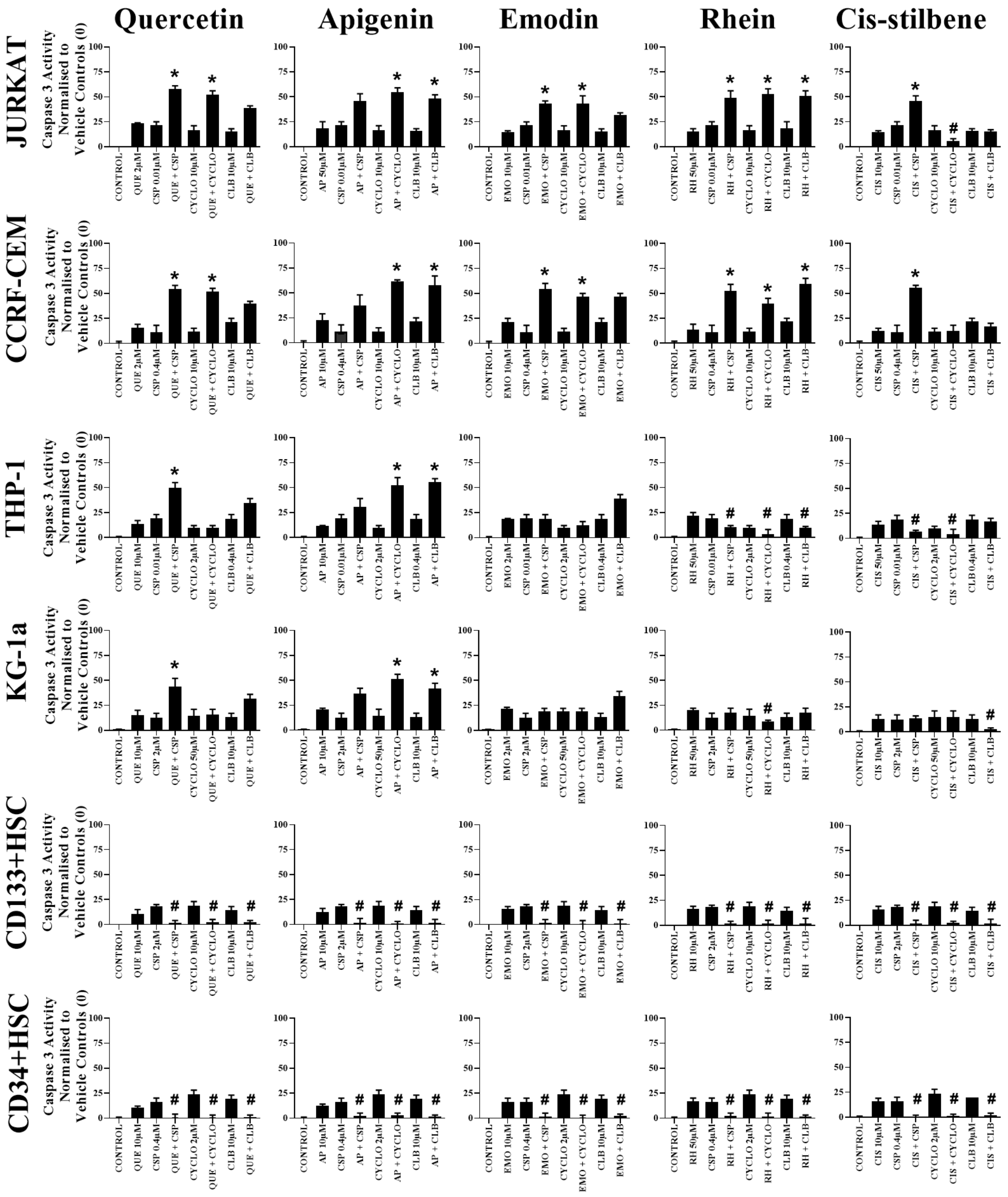

Supplementary Figure 3: The effect of cisplatin (CSP), cyclophosphamide (CYCLO) and chlorambucil (CLB) when used in combination with quercetin (QUE), apigenin (AP), emodin (EMO), rhein (RH), or cis-stilbene (CIS) on caspase 3 activity; in two lymphoid (Jurkat and CCRF-CEM) and two myeloid (THP-1 and KG-1a) leukaemia cell lines, and two non-tumour control cells ($CD133_+$ HSC and $CD34_+$ HSC). This was evaluated by NucView caspase 3 activity assay and flow cytometry. Cells were treated with CSP, CYCLO or CLB and polyphenols alone and in combination for 24 h using their lowest-significant doses (LSD), together with a vehicle control. All data was normalised to the vehicle control, which was assigned 0% apoptosis. The data was expressed as medians with ranges ($N = 4$). Effects of combination treatments were statistically classified as synergistic (*) causing an increase in caspase 3 activity or antagonistic (#) causing a decrease in caspase 3 activity; when compared to vehicle control, drugs alone and expected values of combination treatments. Statistical significant was set at $P \leq 0.05$.

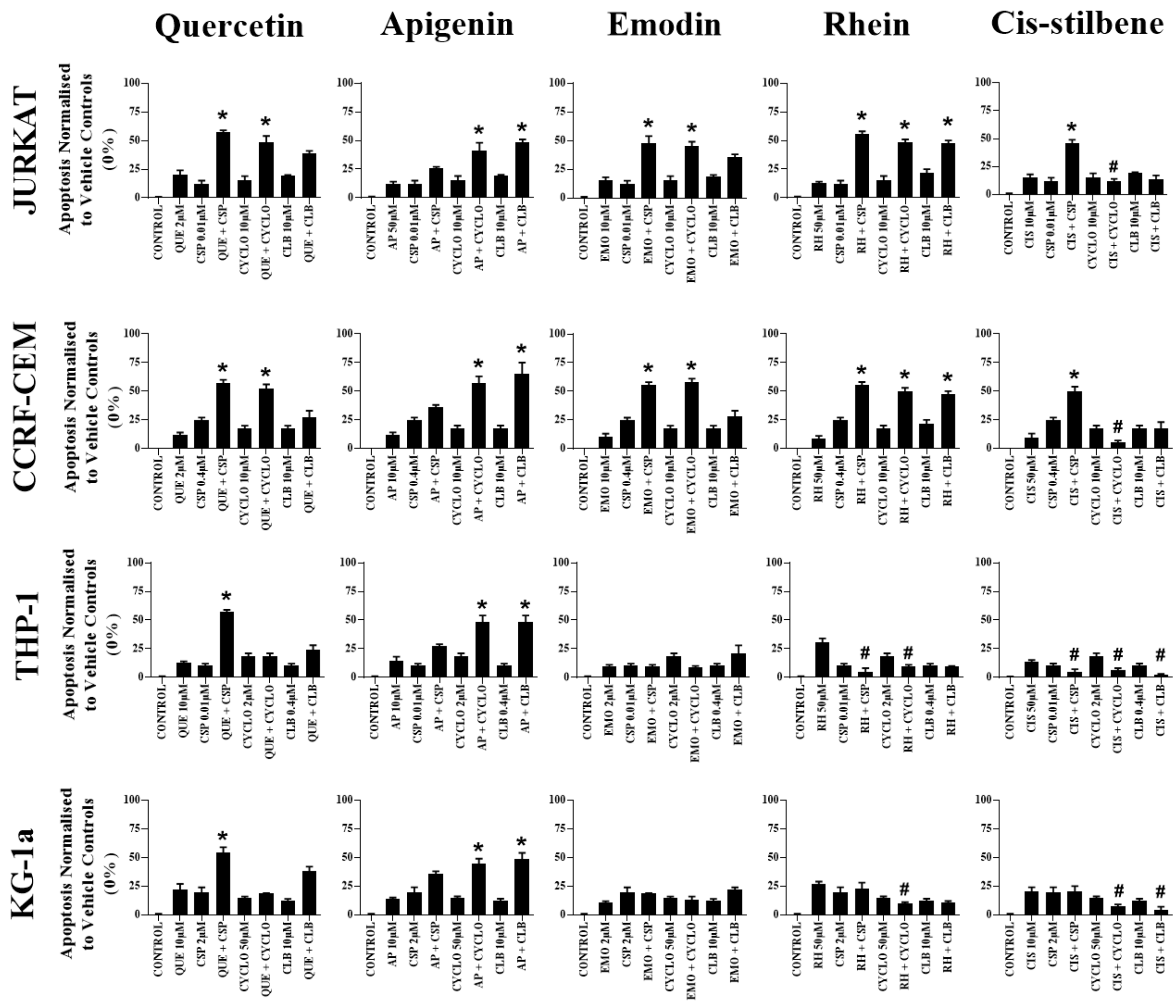

Supplementary Figure 4: The effect of cisplatin (CSP), cyclophosphamide (CYCLO) and chlorambucil (CLB) when used in combination with quercetin (QUE), apigenin (AP), emodin (EMO), rhein, (RH) or cis-stilbene (CIS) on apoptosis morphological changes; in two lymphoid (Jurkat and CCRF-CEM) and two myeloid (THP-1 and KG-1a) leukaemia cell lines. This was evaluated by double staining with Hoechst 33342/PI using fluorescence microscopy. Cells were treated with CSP, CYCLO or CLB and polyphenols alone and in combination for 24 h using their lowest-significant doses (LSD). Data was normalised to the vehicle control, which was assigned 0% apoptosis. The data was expressed as medians with ranges ($N = 4$). Effects of combination treatments were statistically classified as synergistic (*) causing an increase in apoptosis or antagonistic (#) causing a decrease in apoptosis; when compared to vehicle control, drugs alone and expected values of combination treatments. Statistical significant was set at $P \leq 0.05$.

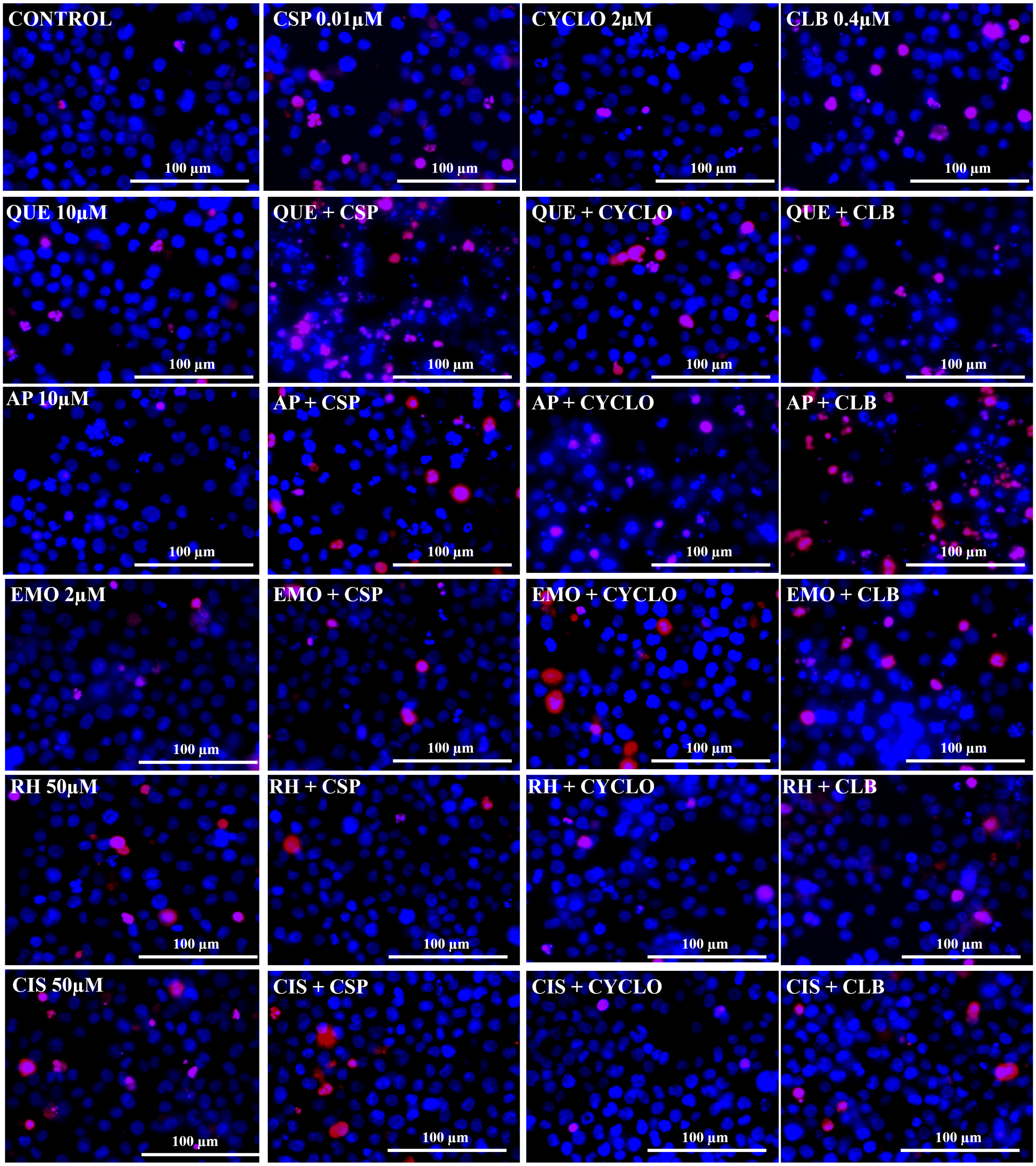

Supplementary Figure 5: An example of morphological assessment of apoptosis using Hoechst 33342/PI for THP-1 myeloid leukaemia cells when treated with lowest-significant doses (LSD) of cisplatin (CSP), cyclophosphamide (CYCLO) and chlorambucil (CLB), and polyphenols (quercetin (QUE), apigenin (AP), emodin (EMO), rhein, (RH) or *cis*-stilbene (CIS)) alone and in combination for 24 h. Apoptotic cells were identified by their intensely bright blue stained nuclei with chromatin condensation, and the formation of apoptotic bodies, compared to normal nuclei which were larger and less intensely stained. Late apoptotic cells and dead cells appear red. Scale bar = 100 µm.

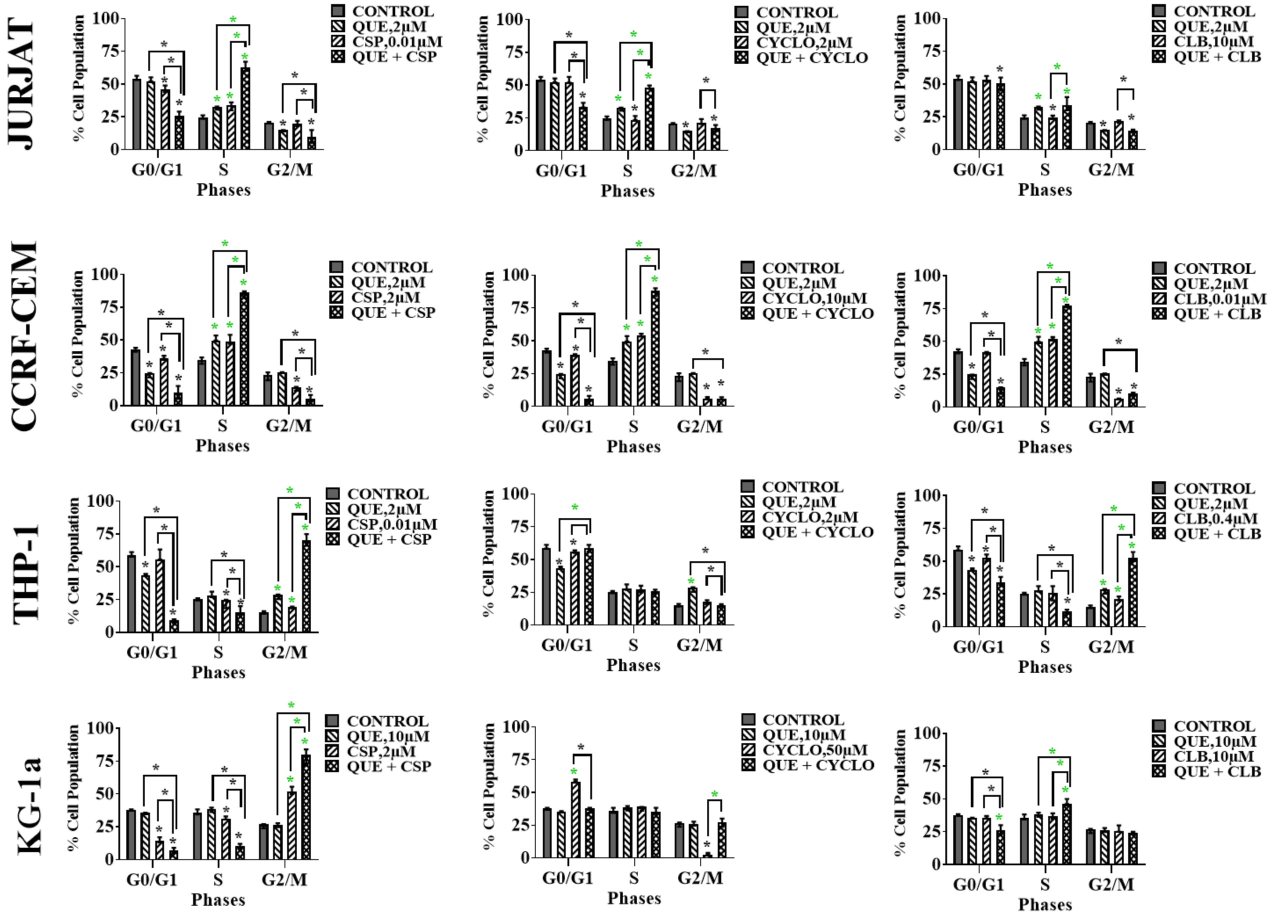

Supplementary Figure 6: The effect of cisplatin (CSP), cyclophosphamide (CYCLO) and chlorambucil (CLB) when used in combination with quercetin (QUE); in two lymphoid leukaemia cell lines (Jurkat and CCRF-CEM) and two myeloid leukaemia cell lines (THP-1 and KG-1a). This was analysed by flow cytometry following propidium iodide staining. Cells were treated with CSP, CYCLO or CLB and quercetin alone and in combination for 24 h using their lowest-significant doses (LSD) as determined by CellTiter-Glo® assay, together with a vehicle control. The percentage of cells in each phase was analysed with FlowJo software using Watson pragmatic model. The data was expressed as medians with ranges (*N* = 4). Statistical significance of combination treatments was determined and compared with the vehicle control and the individual treatments alone. The green asterisk (*) represents significant increase in cell accumulation in a phase of the cell cycle; whilst the black asterisk (*) indicates a significant decrease in cell accumulation in a phase of the cell cycle. Statistical significant was set at $P \leq 0.05$.

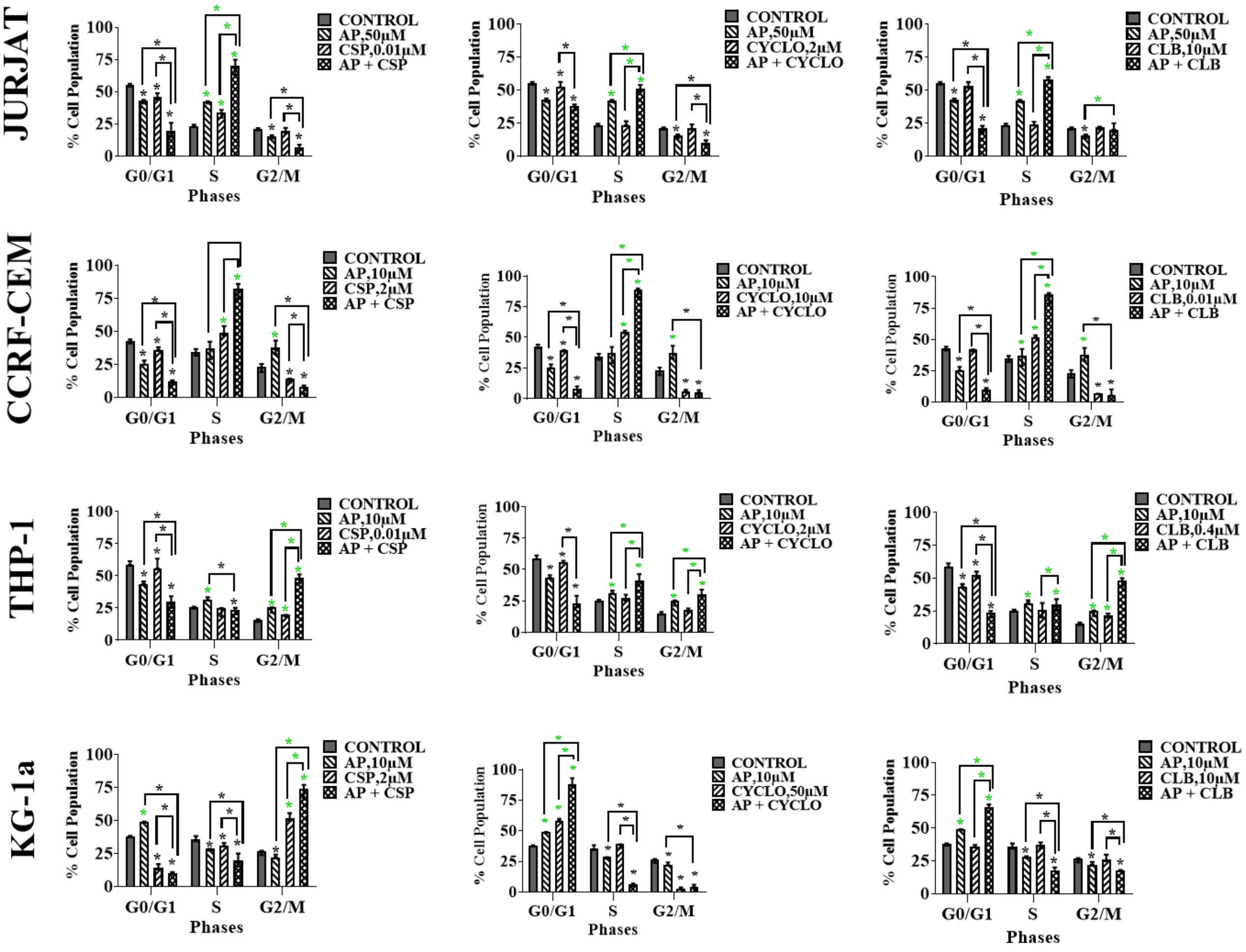

Supplementary Figure 7: The effect of cisplatin (CSP), cyclophosphamide (CYCLO) and chlorambucil (CLB) when used in combination with apigenin (AP); in two lymphoid leukaemia cell lines (Jurkat and CCRF-CEM) and two myeloid leukaemia cell lines (THP-1 and KG-1a). This was analysed by flow cytometry following propidium iodide staining. Cells were treated with CSP, CYCLO or CLB and apigenin alone and in combination for 24 h using their lowest-significant doses (LSD) as determined by CellTiter-Glo® assay, together with a vehicle control. The percentage of cells in each phase was analysed with FlowJo software using Watson pragmatic model. The data was expressed as medians with ranges ($N = 4$). Statistical significance of combination treatments was determined and compared with the vehicle control and the individual treatments alone. The green asterisk (*) represents significant increase in cell accumulation in a phase of the cell cycle; whilst the black asterisk (*) indicates a significant decrease in cell accumulation in a phase of the cell cycle. Statistical significant was set at $P \leq 0.05$.

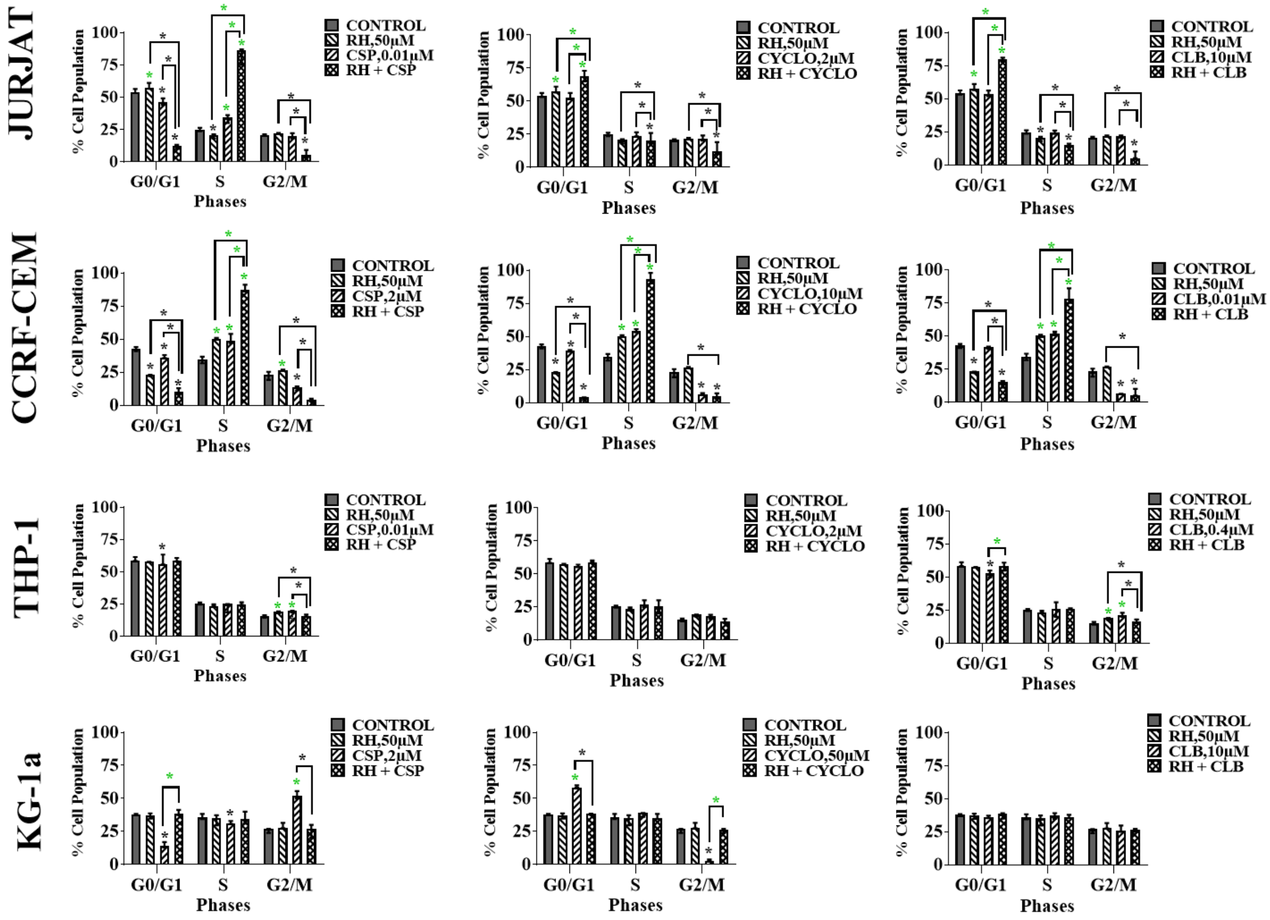

Supplementary Figure 8: The effect of cisplatin (CSP), cyclophosphamide (CYCLO) and chlorambucil (CLB) when used in combination with emodin (EMO); in two lymphoid leukaemia cell lines (Jurkat and CCRF-CEM) and two myeloid leukaemia cell lines (THP-1 and KG-1a). This was analysed by flow cytometry following propidium iodide staining. Cells were treated with CSP, CYCLO or CLB and emodin alone and in combination for 24 h using their lowest-significant doses (LSD) as determined by CellTiter-Glo® assay, together with a vehicle control. The percentage of cells in each phase was analysed with FlowJo software using Watson pragmatic model. The data was expressed as medians with ranges (*N* = 4). Statistical significance of combination treatments was determined and compared with the vehicle control and the individual treatments alone. The green asterisk (*) represents significant increase in cell accumulation in a phase of the cell cycle; whilst the black asterisk (*) indicates a significant decrease in cell accumulation in a phase of the cell cycle. Statistical significant was set at $P \leq 0.05$.

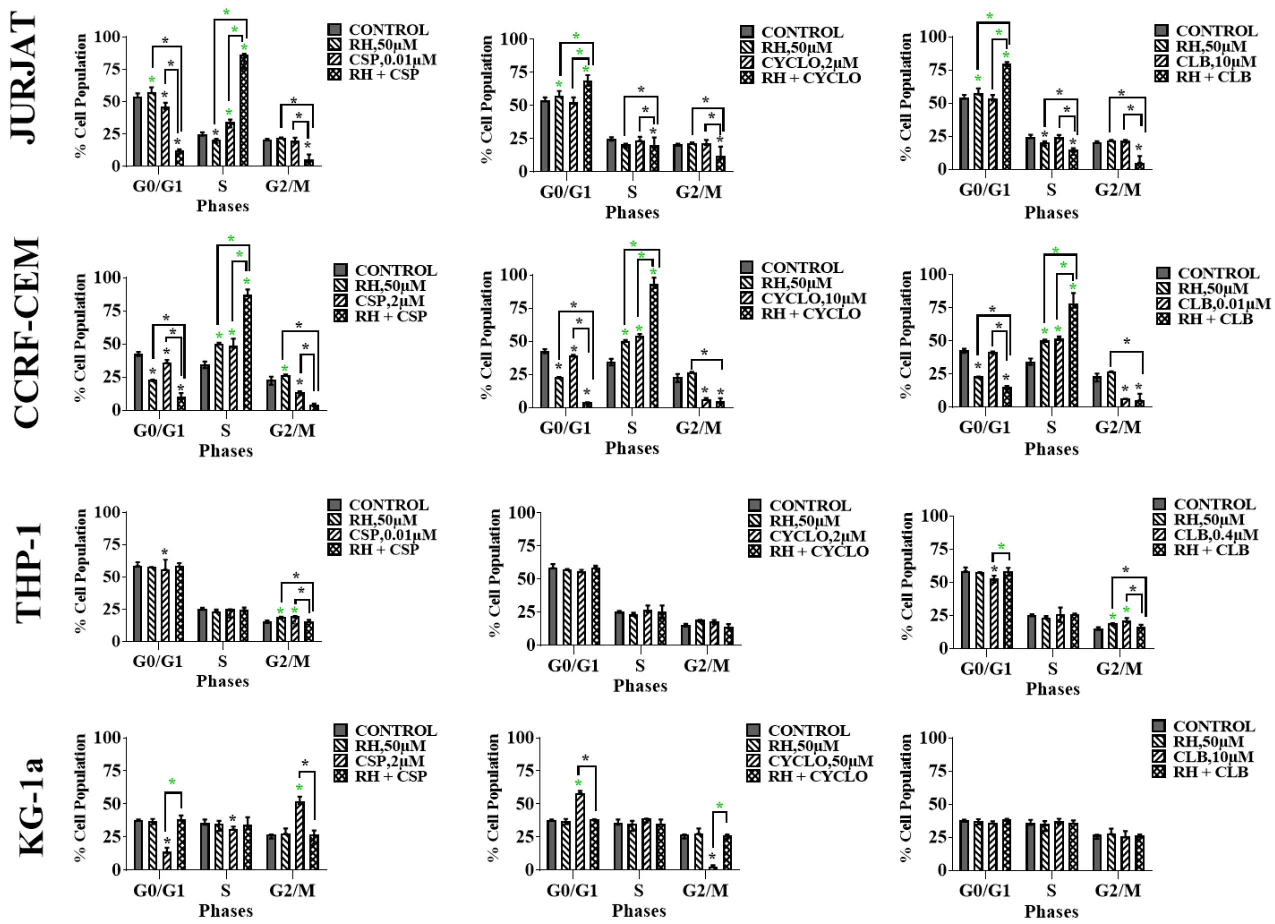

Supplementary Figure 9: The effect of cisplatin (CSP), cyclophosphamide (CYCLO) and chlorambucil (CLB) when used in combination with rhein (RH): in two lymphoid leukaemia cell lines (Jurkat and CCRF-CEM) and two myeloid leukaemia cell lines (THP-1 and KG-1a). This was analysed by flow cytometry following propidium iodide staining. Cells were treated with CSP, CYCLO or CLB and rhein alone and in combination for 24 h using their lowest-significant doses (LSD) as determined by CellTiter-Glo® assay, together with a vehicle control. The percentage of cells in each phase was analysed with FlowJo software using Watson pragmatic model. The data was expressed as medians with ranges (*N* = 4). Statistical significance of combination treatments was determined and compared with the vehicle control and the individual treatments alone. The green asterisk (*) represents significant increase in cell accumulation in a phase of the cell cycle; whilst the black asterisk (*) indicates a significant decrease in cell accumulation in a phase of the cell cycle. Statistical significant was set at $P \leq 0.05$.

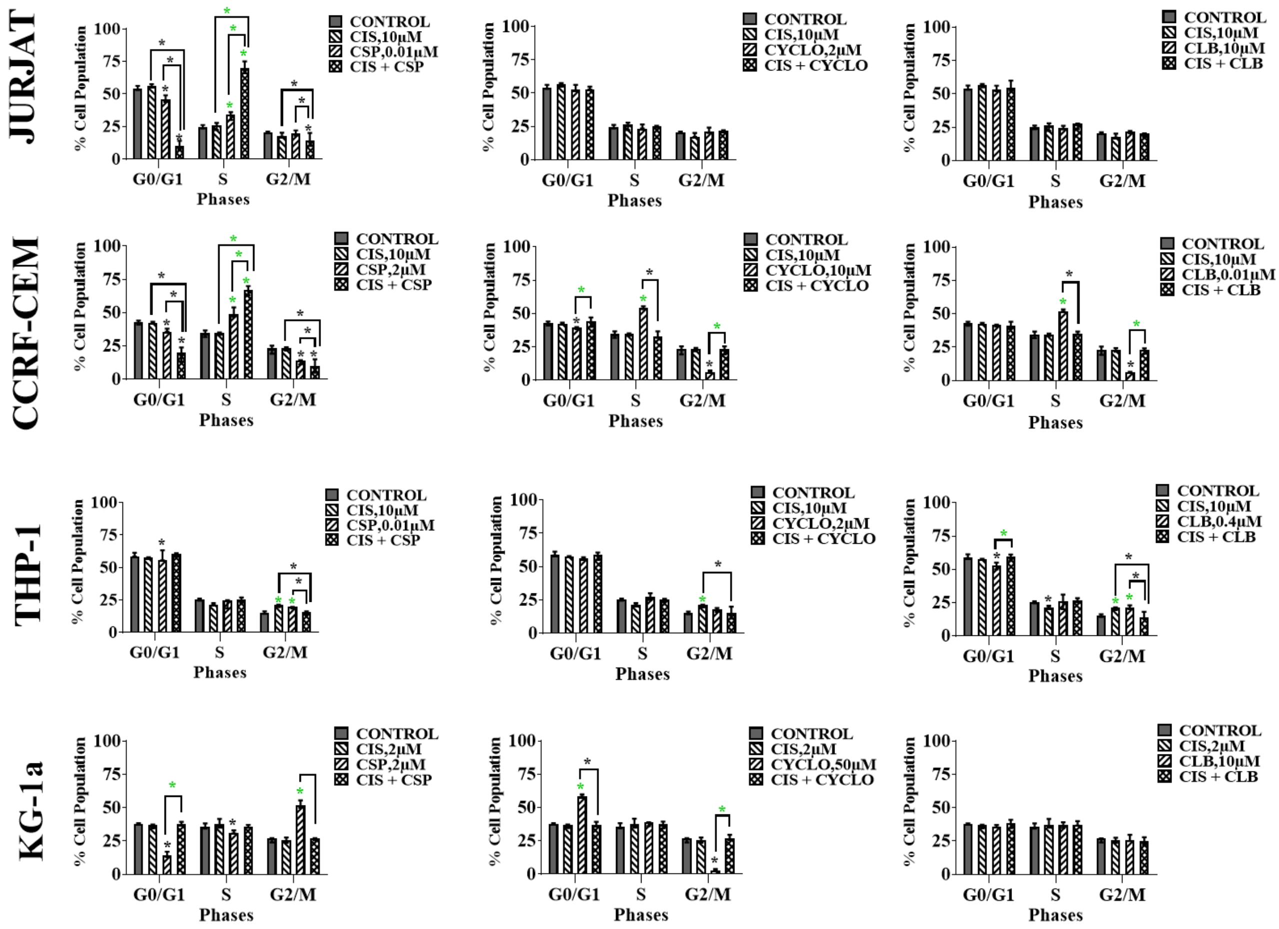

Supplementary Figure 10: The effect of cisplatin (CSP), cyclophosphamide (CYCLO) and chlorambucil (CLB) when used in combination with *cis*-stilbene (CIS): in two lymphoid leukaemia cell lines (Jurkat and CCRF-CEM) and two myeloid leukaemia cell lines (THP-1 and KG-1a). This was analysed by flow cytometry following propidium iodide staining. Cells were treated with CSP, CYCLO or CLB and *cis*-stilbene alone and in combination for 24 h using their lowest-significant doses (LSD) as determined by CellTiter-Glo® assay, together with a vehicle control. The percentage of cells in each phase was analysed with FlowJo software using Watson pragmatic model. The data was expressed as medians with ranges ($N = 4$). Statistical significance of combination treatments was determined and compared with the vehicle control and the individual treatments alone. The green asterisk (*) represents significant increase in cell accumulation in a phase of the cell cycle; whilst the black asterisk (*) indicates a significant decrease in cell accumulation in a phase of the cell cycle. Statistical significant was set at $P \leq 0.05$.

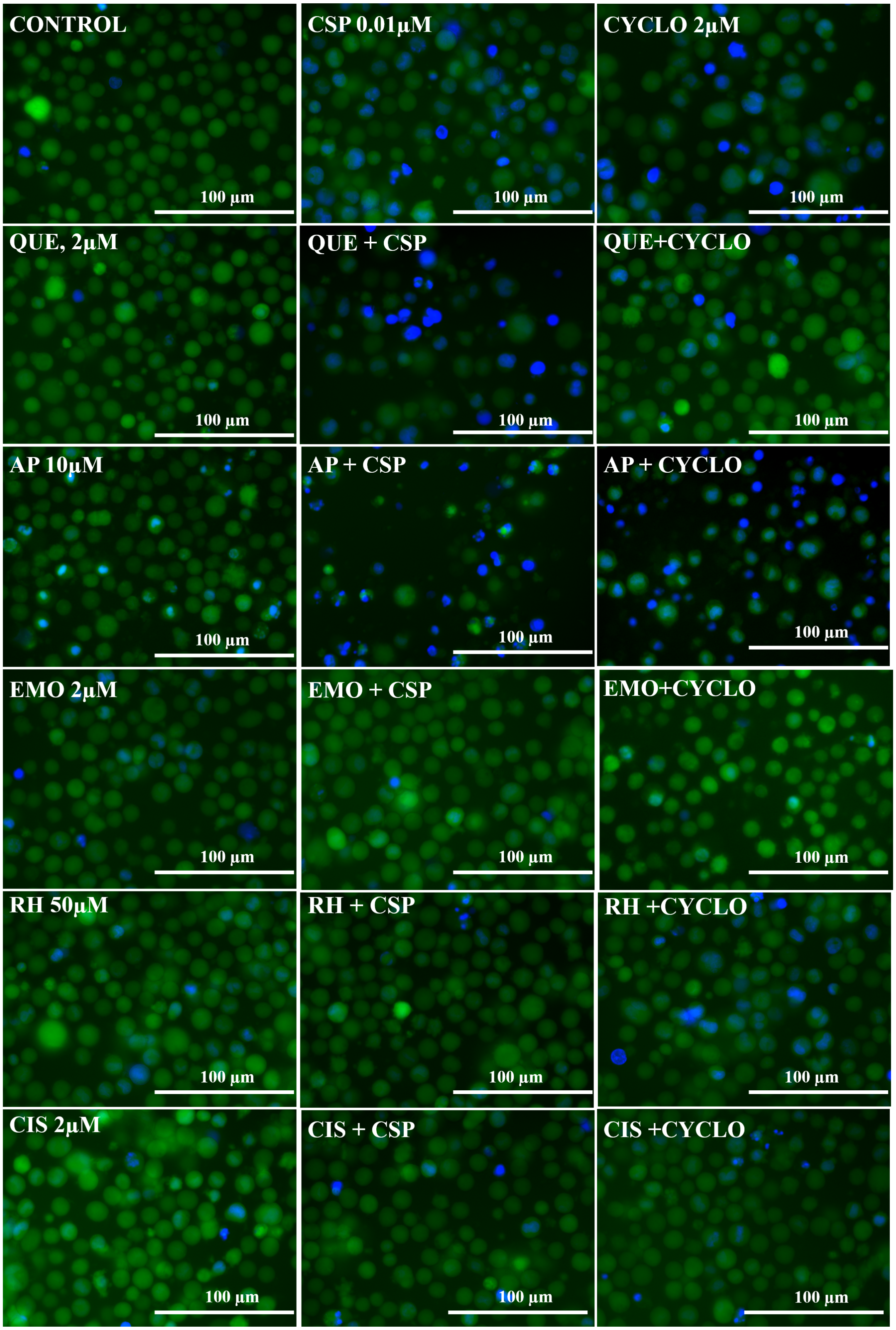

Supplementary Figure 11: An example of fluorescent detection of cellular glutathione (GSH) for THP-1 myeloid leukaemia cells when treated with LSDs of cisplatin (CSP) and cyclophosphamide (CYCLO), and polyphenols (quercetin (QUE), apigenin (AP), emodin (EMO), rhein, (RH) or cis-stilbene (CIS)) alone and in combination for 24 h; using the CellTracker GSH-Green 5-chloromethylfluorescein diacetate (CMFDA) and blue Hoechst 33342 stains. Cells with green-CMFDA staining indicate the presence of GSH localised in the cell cytoplasm and nucleus of only live cells; while cells with blue Hoechst 33342 staining indicate a decrease in GSH localised the nuclei of both living and dead cells. Scale bar = 100 µm.
